# Mixture-Based Screening of Focused Combinatorial Libraries by NMR: Application to the Antiapoptotic Protein hMcl-1

**DOI:** 10.1021/acs.jmedchem.3c01073

**Published:** 2023-07-19

**Authors:** Giulia Alboreggia, Parima Udompholkul, Carlo Baggio, Maurizio Pellecchia

**Affiliations:** Division of Biomedical Sciences, School of Medicine, University of California Riverside, 900 University Avenue, Riverside, California 92521, United States

## Abstract

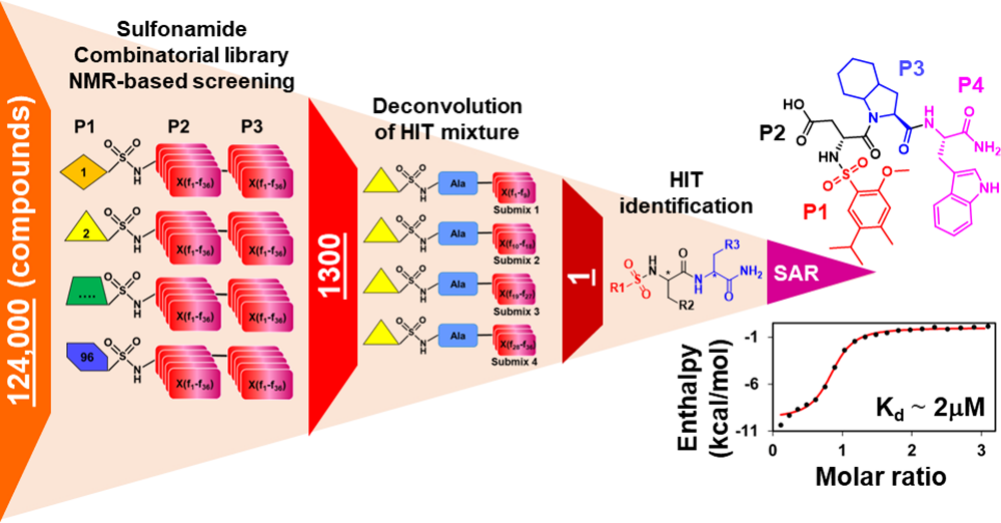

We report on an innovative ligand discovery strategy based on protein NMR-based screening of a combinatorial library of ∼125,000 compounds that was arranged in 96 distinct mixtures. Using sensitive solution protein NMR spectroscopy and chemical perturbation-based screening followed by an iterative synthesis, deconvolutions, and optimization strategy, we demonstrate that the approach could be useful in the identification of initial binding molecules for difficult drug targets, such as those involved in protein–protein interactions. As an application, we will report novel agents targeting the Bcl-2 family protein hMcl-1. The approach is of general applicability and could be deployed as an effective screening strategy for de novo identification of ligands, particularly when tackling targets involved in protein–protein interactions.

## Introduction

Solution NMR spectroscopy is undoubtedly one of the most reliable biophysical approaches for hit validation and for hit identification in fragment-based drug discovery.^1^ Several pharmaceutical companies and academic drug discovery laboratories heavily rely on protein NMR spectroscopy strategies to validate hits that are discovered using other benchtop techniques. In recent years, alternative strategies have been proposed that somewhat were expected to replace NMR in these tasks, including interferometry-based approaches, MS-based strategies, thermodynamic measurements, denaturation thermal shift measurements, or even high-throughput crystallization, to cite a few. However, over the years, the hype that often surrounded these emerging strategies was met with the realization that none of those approaches could provide the reliability of protein NMR spectroscopy to validate the binding of putative hits to a target protein.^1−3^ Some of the drawbacks of NMR spectroscopy, such as the molecular-weight limitation or the intrinsically low throughput of NMR measurements, were also addressed in several ways.^3^ Use of TROSY-NMR^4−6^ and/or selective ^13^C-methyl labeling^7−9^ and deuteration often allows analyzing ligand binding to proteins as large as 100 kDa.^10^ Another possible drawback of NMR screening strategies in drug discovery is the inherently low throughput of NMR experiments. This constraint rendered NMR limited to screening of small compound libraries, generally limited to a few hundred compounds. Typically, in fragment-based campaign screening,^11,12^ such libraries are tested by NMR pooling compounds in mixtures of 3–20 compounds.^1,13,14^ While the approach has been useful in several instances, in recent years, we demonstrated that pooling compounds in mixtures up to thousands of peptide-like agents as in the HTS by NMR (high-throughput screening by nuclear magnetic resonance) approach^15−18^ can be accomplished with libraries arranged in a positional scanning fashion, bringing NMR on par with other techniques with respect to throughput and chemical space sampled in a screening campaign. Recent examples from our laboratory demonstrated that the identification of initial weakly binding tri- or tetrapeptides could lead to optimized agents using traditional structure–activity relationship (SAR) studies and, when supported by structural studies of the complex, introduction of mild electrophiles to reach binding site nucleophilic amino acids such Cys, Lys, or Tyr.^19−21^ Given that the elements of the library have larger molecular weights, hence increased chemical complexity, than typical fragments, the approach seemed suitable for antagonizing protein–protein interactions that involve large binding surface areas.

Here, we report on a novel tripeptide-like combinatorial library designed for NMR-based screening purposes, which is composed of approximately 125,000 compounds, arranged in 96 mixtures of ∼1300 elements each. Like the HTS by NMR, the elements of each mixture are characterized by a common N-terminal fragment coupled to all possible dipeptides from selected 36 natural and non-natural amino acids. However, to decrease the peptide-like nature of the library elements, the N-terminal fragment is linked to the dipeptide via a sulfonamide bond. We describe the strategy adopted to screen the 125,000-compound library by sensitive protein NMR spectroscopy in solution followed by iterative deconvolution and optimization strategies to identify initial binding agents. As an application, we report on the screening, deconvolution, and initial optimization of small molecules binding to the Bcl-2 family protein hMcl-1.

## Results and Discussion

### The Sulfonamide-dipeptide Combinatorial Library

While the identification of initial weakly binding agents to a protein target requires sensitive biophysical approaches, such as NMR spectroscopy, these methods tend to usually present inherently a low throughput compared to typical HTS biochemical screens. To ameliorate this issue and increase the chemical space that can be sampled by NMR, we have recently proposed to prepare compound libraries in mixtures, arranged in a positional scanning (POS) fashion.^15−18,22^ Alternatively, we also proposed to derivatize a POS library with a common binding element for a given target or a class of targets.^15,16,22,23^ Hits within these peptide-like libraries still require significant optimization to obtain potent and selective druglike molecules.^22,24^ To exemplify the library synthesis and the optimization of the resulting hits into more druglike molecules, we propose a new library and an associated deconvolution strategy. To reduce the peptide-like nature of the agents, the first element of the library was chosen among several sulfonyl chlorides that was efficiently reacted with the N-terminus of each element of a mixture of dipeptides (36 × 36 chosen among natural and non-natural aminoamides) using conventional solid-phase synthesis. We chose sulfonamides given the efficiency of the reaction of sulfonyl chlorides with primary amines and the availability at low cost of several fragment-like sulfonyl chlorides. However, other reactions could be envisioned, and several libraries could be built using other functional groups and chemistry.^18,25−33^ Hence, we wish to present our implementation using sulfonyl chlorides, and the strategies used could be easily adaptable to other possible libraries. In this implementation, 96 sulfonyl chlorides were selected based on cost and diversity of elements (Table S1) and coupled each to the 36 × 36 mixture on resin using conventional parallel solid-phase synthesis, using a 10× excess of sulfonyl chloride, and incubating for 2 days. After washing to eliminate unreacted agents and coupling reagents, the mixtures were cleaved from the resin, and the crude was lyophilized three times to eliminate TFA and redissolved in DMSO to obtain stock solutions at a 200 mM total concentration. The library was prepared by InnoPep, Inc. (San Diego). Similarly, submixtures with fewer elements of the dipeptides (see below) were prepared using the same solid-phase protocol.

**Figure 1:**
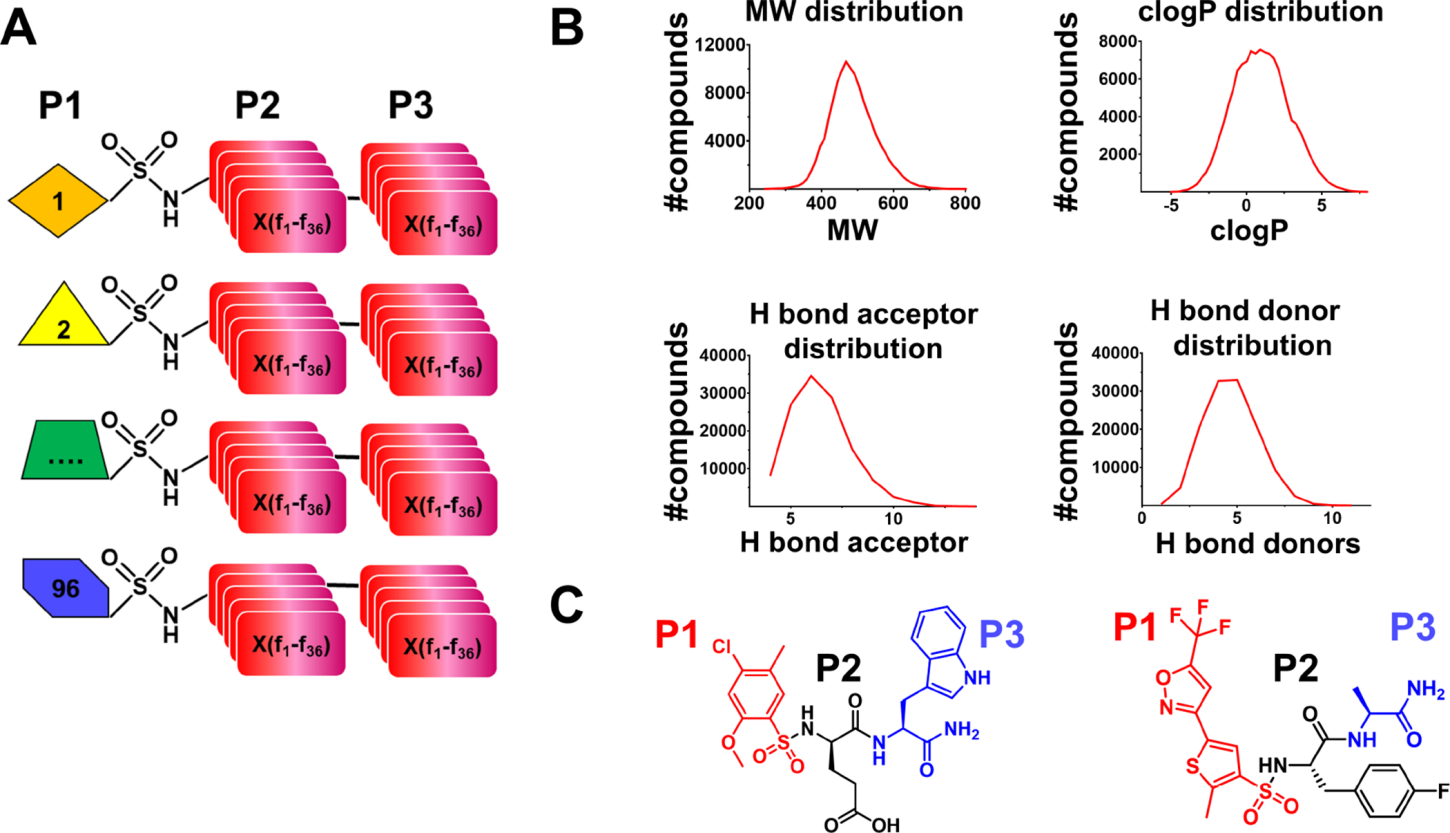
Mixture-based combinatorial library for NMR-based screening. (A) Schematic representation of the library. The library is composed of 96 P1 fragments (Table S1) coupled via a sulfonamide bond to 36 × 36 dipeptides (see also Figure 4A). (B) Critical parameters describing the composition of the library. (C) Representative structures of library elements.

To introduce the randomized P2 or P3 positions, isokinetic mixtures of Fmoc-amino acids were preactivated with HATU and Oxyma Pure in DMF (2 mL). The solutions were added to the resins and agitated for 2 h. The mixtures were prepared considering the mol % of each amino acid (Table S2) or analogues of amino acids; the most reactive amino acids were used as 3 equiv, while an increased number of equivalents were used for the other amino acids (see methods).^27,31^ A schematic illustration of the library is reported in Figure 1, where critical physical–chemical properties of the library are also reported.

### Application to the Discovery of hMcl-1 Binding Agents

The design of potent and selective ligands that target protein–protein interactions (PPIs) remains one of the most challenging tasks in drug discovery despite PPIs currently representing one of the most abundant classes of therapeutically viable targets. Intriguingly, the only rationally designed FDA-approved drug targeting a PPI is the Bcl-2 targeting agent Venetoclax,^34^ derived using the SAR by NMR approach^12,35^ and extensive follow-up structure-based optimizations.^11,34^ While initial fragment hits (low-molecular-weight molecules with low affinity) can be usually identified using NMR,^3^ the optimization of these into potent and selective agents remains an often unsurmountable challenge. Strategies that can be efficiently used iteratively to monitor ligand binding unambiguously and that can also provide qualitative yet meaningful information on relative binding affinity, off-rates, or induced conformational changes are critical to aid these challenging endeavors.

As an application to our novel screening library, we tested it against hMcl-1, another member of the Bcl-2 family protein. As mentioned above, to target PPIs, we have recently reported the HTS by NMR approach^17,18^ that consists of combining principles of positional scanning combinatorial chemistry^31^ and protein-based NMR screening to identify, within libraries of >100,000 compounds, possible initial binders to protein–protein interactions. More recently, we also proposed deploying a “focused” positional scanned library (*f*HTS by NMR) for the approach, in which each element of the library is designed to be derivatized with a target-specific (or a target class-specific) chemical binding moiety.^22,23,36^ Using the HTS by NMR against EphA4-LBD, we identified initial ligands of weak affinity (>300 μM) that, nonetheless, using either the *f*HTS-by-NMR and/or iterative SAR optimizations, including the X-ray structure of the complex, could be optimized into potent and selective, low nanomolar affinity agents.^15,16,22−24^ Hence, deriving initial bona fide binding agents (as detected by sensitive protein-based NMR ligand binding assays) represents an exceptionally valuable starting point for the design of potent agents, especially if later supported by high-resolution structural information of the complex.

**Figure 2:**
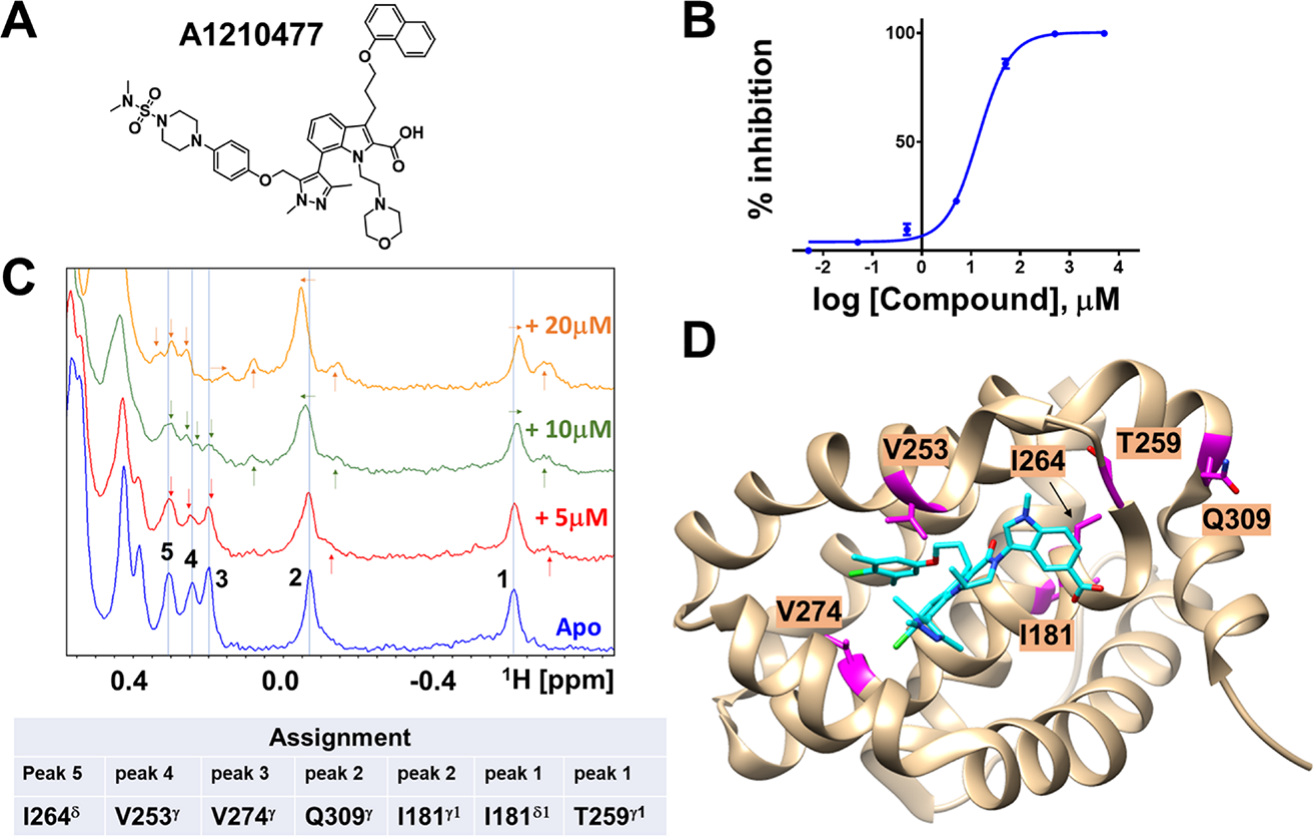
NMR-based screening method for hMcl-1. (A) Chemical structure of the potent reference ligand A1210477. (B) Dose–response curve for A1210477 displacement of the binding between hMcl-1 and a BH3 peptide in a DELFIA assay (see methods). The IC_50_ value of 14 nM agrees with the value reported in the literature (LE of ∼0.17). (C) Aliphatic region of the 1D ^1^H NMR spectra of hMcl-1 (20 μM) recorded in the absence (blue) and presence of various concentrations of A1210477 as indicated. The resonance assignment is also reported. Vertical blue lines highlight residues in the binding site that are visibly perturbed by the ligand and hence are used to rank order test mixtures and compounds. The arrows highlight the numerous changes/shifts in the 1D ^1^H NMR spectra of hMcl-1 caused by the presence of A1210477. (D) Ribbon-and-stick representation of the complex between hMcl-1 and a ligand analogue to A1210477 (PDB ID 6NE5). Binding site residues that are visible in the 1D ^1^H aliphatic spectrum of hMcl-1 are also displayed and labeled.

As we demonstrated in several recent publications, a sensitive and less prone to false positive, label-free NMR-binding assay consists of observing the 1D ^1^H aliphatic region of the protein target (hMcl-1) in the region below 1 ppm, given that it is typically void of signals of small molecules from the library (Figure 2).^3^ Hence, simple 1D ^1^H NMR screening collected in the absence and presence of test ligands or mixtures of test ligands can be used to efficiently identify putative binders. Perturbations observed by ligand binding can be measured and qualitatively used to roughly rank order compound binding, or compound mixtures in this case, in the initial screening campaign (Figure 2). As a control, we used a potent hMcl-1 binding ligand A1210477^37^ (Figure 2A). Using our previously developed DELFIA displacement assay,^38^ we could verify that the agent is effective in displacing a biotinylated BH3 peptide from hMcl-1 with an IC_50_ value of 14 nM that agrees with the reported affinity for this agent (Figure 2B).^37^ Exposing the ligand to hMcl-1 causes perturbation in its aliphatic region of the 1D ^1^H NMR spectrum, where several binding site residue resonances occur (Figure 2C,D), suggesting that the assay could be used to effectively identify hMcl-1 binding agents.

**Figure 3:**
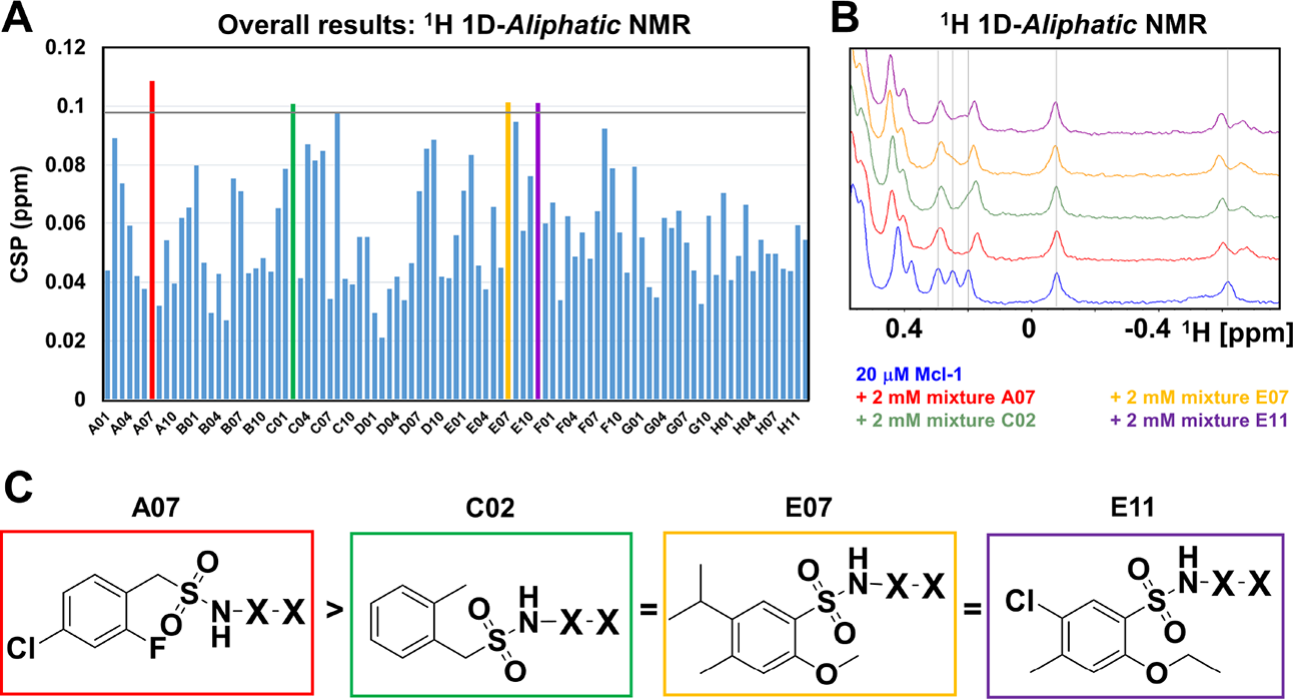
Summary illustration of the screening results for hMcl-1. (A) Plot of the observed average chemical shift perturbations for the peaks highlighted in panel B, as a function of the P1 position of the tested mixtures. (B) Representative 1D ^1^H NMR spectra of hMcl-1 in the absence or presence of selected positive mixtures. (C) Chemical structures and rank ordering of the P1 fragment present in positive mixtures. X-X indicates all possible dipeptides comprised by the 36 amino acids in positions P2 and P3.

**Figure 4:**
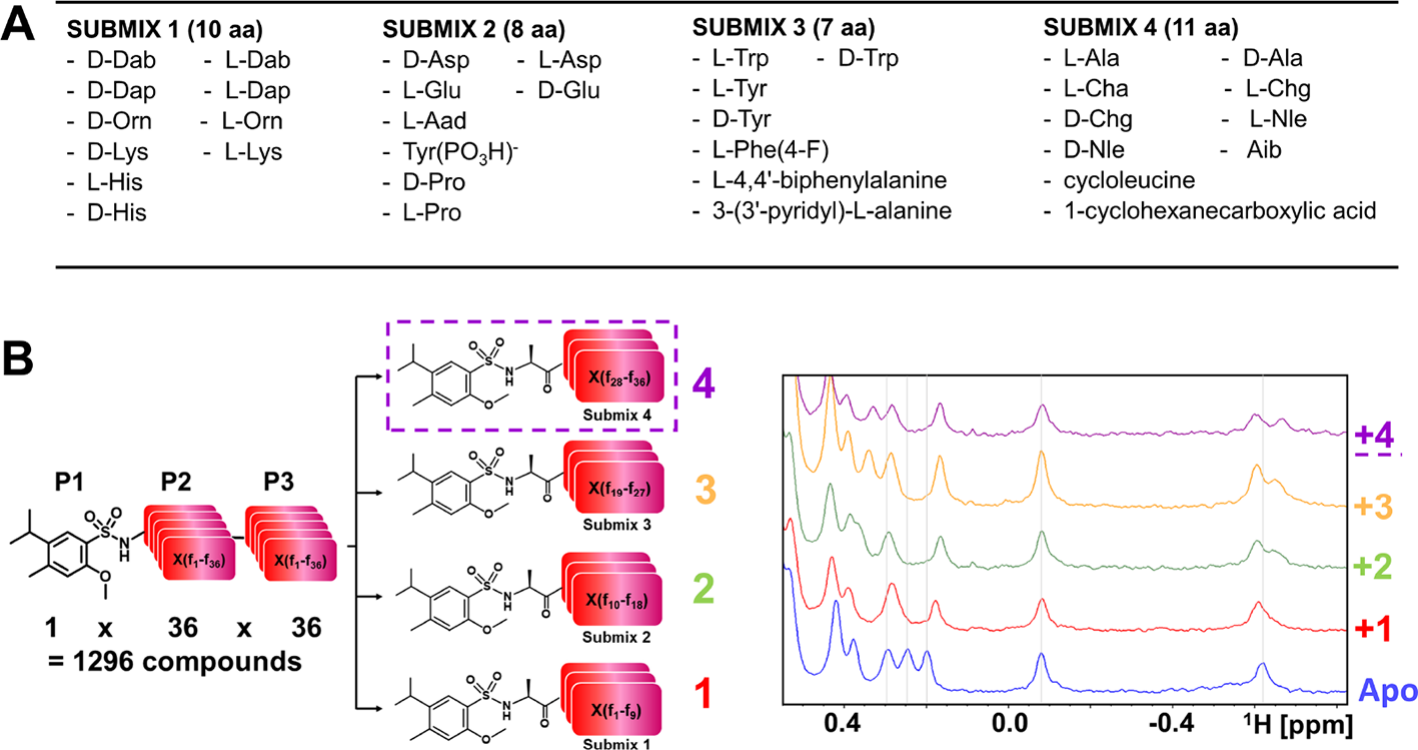
Schematic illustration of the deconvolution process and application to hMcl-1. (A) Composition of the 4 submixtures representing the 36 library elements in positions P2 and P3. The mixtures are grouped by general properties of the side chains (positively charged, negatively charged, aliphatic, and aromatic). To balance the number of elements in each submixture, l-Pro and d-Pro have been grouped with negatively charged amino acids. (B) Illustration of the submixtures synthesized for hMcl-1 following the selection of the reported P1 elements based on the primary screening and relative 1D ^1^H NMR spectra for hMcl-1. The data suggested that submixture 4 may contain the most active P3 element.

Hence, we tested our sulfonamide combinatorial library by exposing hMcl-1 (20 μM) to each of the 96 mixtures (at a 2 mM total concentration) by collecting simple 1D ^1^H NMR spectra and analyzing the aliphatic region of the spectra. At first, it may be counterintuitive to assume that the approach would be able to detect weak binders in mixtures given that the individual compounds have a theoretical concentration of only ∼1.5 μM, while the protein in the NMR assay is at 20 μM in this example. To understand the ability of the approach to detect putative positive hit mixtures under such experimental conditions, one must consider that all compounds in each mixture have the exact same P1 element, and therefore, the concentration of a given element P1 is 2 mM. These elements are likely to be very weak in isolation to be detected even by NMR, but additional interactions can be provided by elements in P2 and P3 that would increase the affinity of the P1 to the detection limits of NMR (triple-digit μM or low mM). The power of the approach is to use protein NMR as detection in which perturbations can be observed by multiple analogue compounds in the mixture, all having a common P1 and perhaps slight variations of P2 and P3 elements. For example, if Val is the best binder in P2, likely other compounds with an aliphatic side chain (see also later Figure 4A to better appreciate this point) could have some affinity for the target, and all cumulatively would cause detectable perturbations in the NMR spectra, having a much greater concentration than the single isolated hit compound. This can be clearly appreciated in the stepwise deconvolutions illustrated below. The data relative to the primary screening campaign of the 96 mixtures are summarized in Figure 3.

We chose mixtures E07 and E11 for the follow-up deconvolution strategies, identifying the reported sulfonamides as possible preferred P1 position elements. Therefore, the next challenge was to identify which library elements in the P2 and P3 positions were better poised to bind hMcl-1 when coupled to these selected P1 elements. The approach that we deployed is schematically illustrated in Figure 4 (see also Figure S1). Briefly, the strategy consisted of synthesizing and testing by NMR 4 lower-complexity mixtures in one position, fixing the identified P1 element and Ala in either position P2 or P3 (Figure 4). The four submixtures are grouped by general properties of the side chains (positively charged, negatively charged, aliphatic, and aromatic; Figure 4A). After selection of the NMR positive submixture, individual compounds are synthesized and tested.

**Figure 5:**
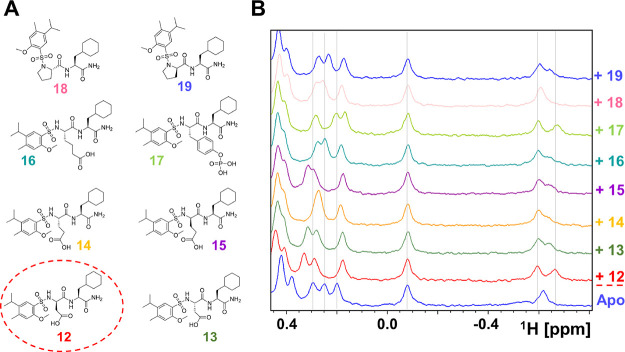
Data relative to the second deconvolution to identify the optimal P2 position element. (A) Chemical structures of the individual compounds synthesized comprising the P1-sub-Mix2-l-Clyclohexyl-Ala that was identified by NMR testing (Figure S3). (B) 1D ^1^H NMR spectra of hMcl-1 recorded in the absence (blue, 20 μM) and presence of the indicated agents at 250 μM.

Finally, agents from submixtures inducing the largest chemical shift perturbations are individually synthesized and tested. The data resulted in the selection of l-cyclohexyl-Ala in P3 (Figure S2). After the P3 position was selected, the procedure was repeated to identify the optimal element in the P2 position among the 36 amino acids. This could have been accomplished either by directly synthesizing and testing all 36 possible agents or by first preparing 4 lower-complexity mixtures, as in this case (Figure S3), followed by testing and synthesis of the individual compounds in the positive submixtures (Figure 5).

In summary, by using this relatively simple sequential approach, in principle, a hit compound out of the 125,000-compound library can be identified by screening the 96 mixtures followed by synthesis and testing of 4 mixtures, and up to 11 individual compounds, for each of the two P2 and P3 positions, are hence attainable without an insurmountable amount of time and resources. Applied to hMcl-1, this strategy converged in compound **12** reported in Figure 5.

**Figure 6:**
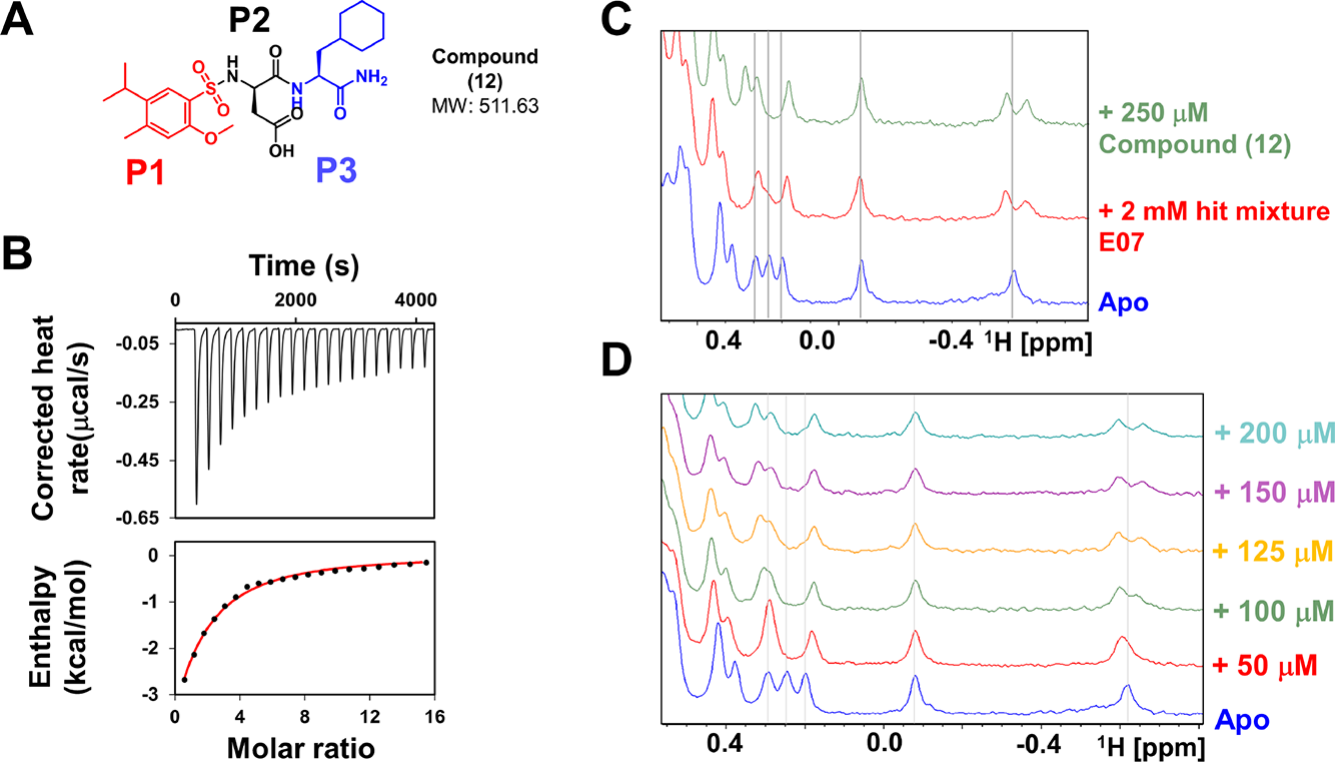
Summary of screening results. (A) Chemical structure of compound **12**. (B) Isothermal titration calorimetry data with compound **12** and hMcl-1. The titration is enthalpy driven and results in a *K*_d_ of ∼450 μM. (C) Comparison of the 1D ^1^H NMR spectra of hMcl-1 recorded in the presence of either the original hit mixture or compound **12** at the indicated concentrations. (D) 1D ^1^H NMR titration spectra of hMcl-1 (20 μM) recorded in various concentrations of compound **12**.

NMR experiments with compound **12** revealed an estimated dissociation constant in the triple-digit micromolar, which was also confirmed by isothermal titration calorimetry (Figure 6). Hence, the screening of 125,000 compounds resulted in the identification of a validated hit with a reasonable affinity, also considering the nature of the target.

To rule out the possibility that the strategy would miss numerous hits as false negatives (or agents that may fall below the limit of detection of the assay under the experimental conditions adopted), we also performed the same deconvolution strategy on a mixture that resulted in negative from the primary screening of the 96 mixtures (Figure S4). While some very small shifts are still observable in NMR spectra of hMcl-1 when exposed to these submixtures of this negative hit, likely attributable to weaker interacting side chains, they are still of much smaller magnitude compared to those reported above for the positive mixture (Figure S4).

### Structure–Activity Relationships and Initial Optimization Strategies

After SAR studies on compound **12** (Table S3), we thought to further enhance the binding affinity of the compound by elongating the molecule at the C-terminus with a fourth element. We proved this strategy recently in the design of potent agents targeting other PPIs involving the receptor tyrosine kinase EphA4 and its ephrin ligand.^17,22,24^

Hence, additional agents were synthesized and tested in which compound **12** was elongated with each of the 4 submixtures as reported in Figure 4 (see also Figure S5). The data identified that an aromatic amino acid in position 4 could enhance the affinity of the ligand; hence, individual agents were prepared and tested by NMR (Figure S5). This first optimization step identified agent compound **21** that presented a d-Trp at the C-terminus of compound **12** (Figure S6). NMR titration data suggested a dissociation constant for compound **21** of about 30 μM for hMcl-1 (Figure S7).

**Figure 7:**
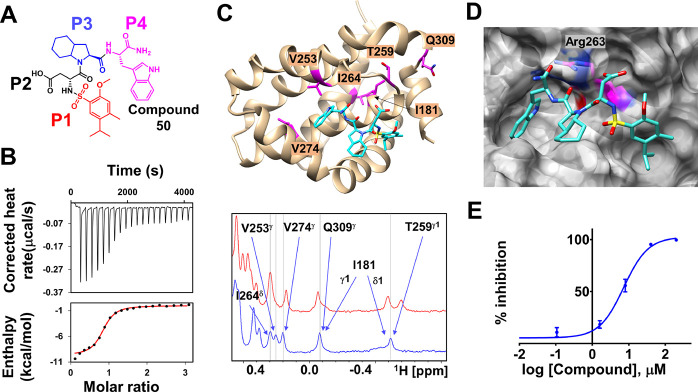
Characterization of final hit compound **50**. (A) Chemical structure of compound **50**. (B) Isothermal titration calorimetry data for the binding of compound **50** to hMcl-1. Δ*H* = −10 kcal/mol; *K*_d_ = 1.71 ± 0.3 μM (*n* = 2; LE of ∼0.16). (C) Ribbon representation of the docked structure of hMcl-1 in complex with compound **50**. The monitored residues using 1D ^1^H NMR (bottom panel) are highlighted. (D) Same as in (C) but with hMcl-1 in surface representation and highlighting residues Arg 263. (E) DELFIA dose–response curve for compound **50** displacement of a biotinylated-BIM peptide from hMcl-1. IC_50_ = 7.5 μM. Molecular models were prepared with Sybyl (Certara) and analyzed using MOE 2019.0101 (Chemical Computing Group) or Chimera (http://www.cgl.ucsf.edu/chimera).

Finally, SAR studies on compound **21** consisted of the synthesis and iterative evaluations of agents with modifications in P4 with Trp analogues, in P2 with cyclohexyl Ala analogues, and with analogues of P1 (Table S4). The addition of a P5 position was also probed but did not result in significant improvement in binding affinity. These studies are summarized in Table S4 and resulted in the selection of compound **50** (Figure 7A), with a binding affinity of hMcl-1 of ∼2 μM by isothermal titration calorimetry (Figure 7B). Moreover, and in agreement with ITC data, in the DELFIA displacement assay, the compound displayed an IC_50_ value of 7.5 μM against hMcl-1 (Figure 7E), but it was inactive against the more closely related Bcl-2 family protein, namely, hBfl-1.^39,40^

A simple docking strategy was also employed to gather a possible binding pose for the agent in the BH3 binding pocket of hMcl-1 (Figure 7C,D). The obtained pose is reasonable as it positions the agent’s P1 and P4 moieties in two adjacent hydrophobic pockets, while the Asp acid is engaged in a salt bridge with Arg 263, hence binding in a typical fashion as reported for other BH3 mimetics (Figure 7).^38,41−45^

**Figure 8:**
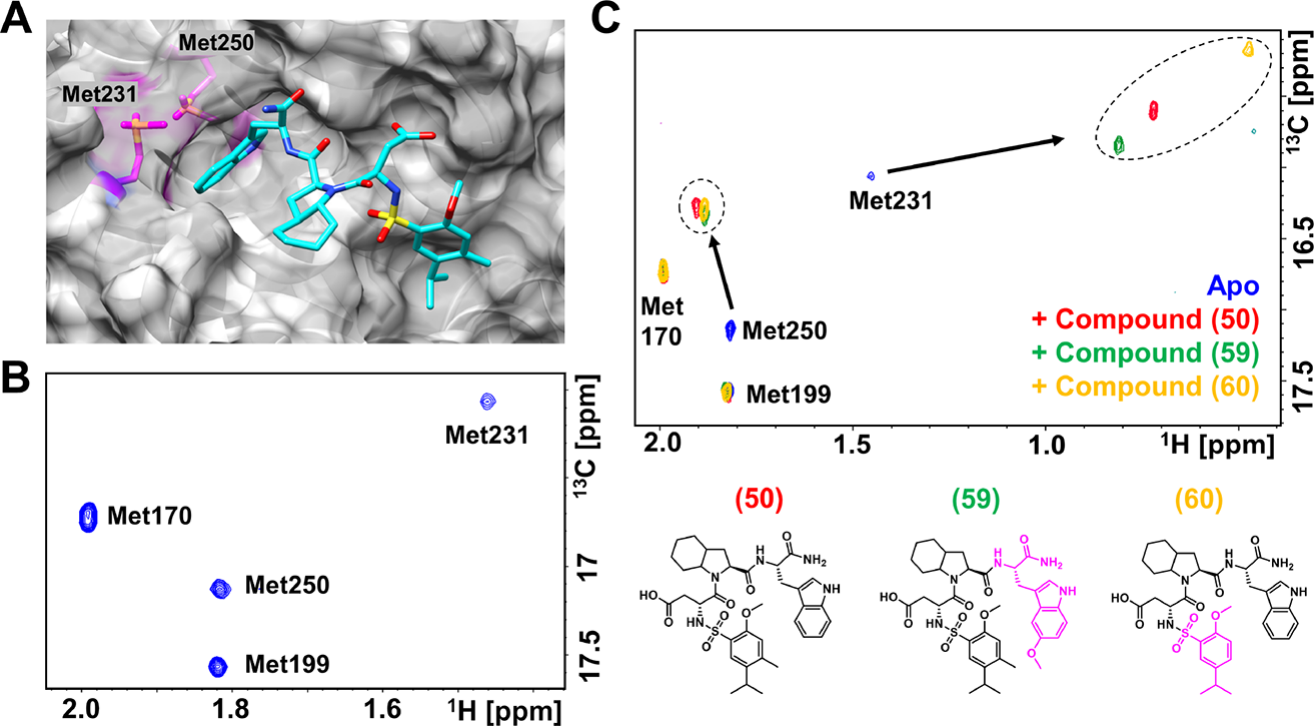
Chemical shift mapping of compound **50** and related using ^13^C,^1^H correlation spectra and selective Met labeling. (A) Docked geometry of compound **50** in the binding site of hMcl-1 (surface representation; PDB ID used for docking was 6P3P). Residues Met 231 and Met 250 are highlighted. Molecular models were prepared with Sybyl (Certara) and analyzed using MOE 2019.0101 (Chemical Computing Group) or Chimera (http://www.cgl.ucsf.edu/chimera).

To further corroborate the possible binding location and pose of the agent, we collected NMR experiments with a selectively ^13^C^ε^-Met-labeled hMcl-1.^1^ In the sequence of hMcl-1, there are 4 Met residues, where two located outside the biding pocket, namely, Met 170 and Met 199, while two Met residues are directly present in the BH3 binding pocket, namely, Met 231 and Met 250 (Figure 8). Hence, we used 2D [^13^C,^1^H] correlation spectra with^13^C^ε^-Met-labeled hMcl-1 and monitored chemical shift perturbations induced by compound **50** and other related ligands, compounds **59** and **60** (Figure 8) (*K*_d_ = 4.62 ± 0.37 (*n* = 2) and 2.84 ± 0.67 μM (*n* = 2), respectively; Figure S8). Here, as expected, very large ligand-induced shifts were observed for the^13^C^ε^/^1^H^ε^ resonances of residue Met 231, presumably due to the ring current effect of the indole ring of the agent, as per the binding pose (Figure 8). Smaller yet still relatively large perturbations were also observed in the resonances of Met 250, located also across the indole in our model but not as directly juxtaposed as Met 231 with our ligand (Figure 8). On the contrary and again in agreement with the binding of the compound in the BH3 binding region, no appreciable chemical shift perturbations were observed in residues Met 170 and Met 199, located well outside the BH3 binding pocket. Interestingly, titration experiments with compound **50** and ^13^C^ε^-Met-hMcl-1 revealed that the perturbations induced on the Met chemical shifts were in slow exchange in the NMR timescale (Figure S9), resulting in an estimated *k*_off_ < 519 s^–1^. Assuming a diffusion limited on the rate of 10^9^ M^–1^ s^–1^, such an off-rate would correspond to a low micromolar dissociation constant (Figure S9). This piece of data supports the location of the binding of our agent and possibly also corroborates its predicted binding pose.

## Conclusions

While in recent years, there have been tremendous advances in drug discovery research for challenging drug targets, targeting PPIs remains difficult with only few approved drugs reported to date, although PPIs represent in principle a large, pharmacologically viable target space. Our recent attempts to identify novel chemotypes targeting PPIs include the use of positional scanning in the HTS by NMR approach and its variation in which one library element is a fixed known binding element as in the focused HTS by NMR approach (*f*HTS by NMR). Both strategies have proven successful in de novo identification on potent ligands against PPIs. Likewise, we also previously reported that an HTS by NMR screening campaign against hMcl-1 of a tetrapeptide library arranged in a positional scanning fashion resulted in a novel Asp-Trp-Asp-Trp tetrapeptide motif of weak affinity.^17^ However, optimization of weak-affinity peptides into potent, druglike agents is notoriously not a trivial task. To simplify such a strategy, we propose here a simpler mixture-based approach that allows for a more immediate and practical preparation of large compound collections that are amenable to screening and deconvolution using protein-based NMR methods, which are less prone to false positive hits. In our application, a validated (by means of NMR spectroscopy and ITC), low micromolar agent with reasonably low molecular weight and ample possibilities for optimizations was identified, representing in principle a novel chemotype targeting hMcl-1. The approach is relatively simple and could find general applicability in a variety of scenarios, including, for example, the creation of libraries of cyclic peptides,^28,46−59^ or similar to the *f*HTS by NMR, for the optimization of an initial binding scaffold identified by other methods.

## Experimental Section

### General Chemistry

All reagents and solvents were obtained from commercial sources, including the majority of Fmoc-protected amino acids and resins for solid-phase synthesis. NMR spectra in DMSO-*d*_6_ were used to evaluate the concentration of stock solutions and were recorded on a Bruker Avance III 700 MHz equipped with a TCI cryoprobe. ^1^H NMR measurements in assay buffer were used for quality control and to routinely verify the solubility of the compounds used for all studies. High-resolution mass spectral data were acquired on an Agilent 6545 Q-TOF LC/MS instrument (Table S4). RP-HPLC purifications were performed on a JASCO preparative system equipped with a PDA detector and a fraction collector controlled by a ChromNAV system (JASCO) on an XTerra C18 10 μm 10 × 250 mm^2^ (Waters). The purity of tested compounds was assessed by HPLC. All compounds had a purity of ≥95% (Figures S10–S12).

### Peptide Synthesis

All agents were synthesized in house by standard solid-phase Fmoc peptide synthesis protocols on Rink amide resin. For each coupling reaction, 3 equiv of Fmoc-AA, 3 equiv of HATU, 3 equiv of Oxyma Pure, and 5 equiv of DIPEA in 1 mL of DMF were used. The coupling reaction was allowed to proceed for 1 h. Fmoc deprotection was performed by treating the resin-bound peptide with 20% piperidine in DMF twice (5 and 20 min of reaction). Peptides were cleaved from Rink amide resin with a cleavage cocktail containing TFA/TIS/water (94:3:3) for 3 h. The cleaving solution was filtered from the resin and evaporated under reduced pressure.

### Compound **12**: (*R*)-4-(((*S*)-1-Amino-3-cyclohexyl-1-oxopropan-2-yl)amino)-3-((5-isopropyl-2-methoxy-4-methylphenyl)sulfonamido)-4-oxobutanoic Acid

Rink amide resin was used as a solid-phase support (0.05 mmol scale). Rink amide resin was Fmoc-deprotected, as described in the previous section, and washed three times with DMF, three times with DCM, and again three times with DMF. Peptide elongation, coupling Fmoc-Cha-OH and Fmoc-d-Asp-OH, and Fmoc deprotection followed standard procedures described in the previous section. For the last reaction, 3 equiv of 5-isopropyl-2-methoxy-4-methylbenzenesulfonyl chloride (sulfonyl chloride of the E07 mixture), 2 mg of DMAP, and 5 equiv of DIPEA in 1 mL of DMF were used. The reaction was left shaking overnight at room temperature. After cleavage, the crude was purified by preparative RP-HPLC using an XTerra C18 (Waters) and a water/acetonitrile gradient (5–100%) containing 0.1% TFA.

### Compound **21**: (*R*)-4-(((*S*)-1-(((*R*)-1-Amino-3-(1*H*-indol-3-yl)-1-oxopropan-2-yl)amino)-3-cyclohexyl-1-oxopropan-2-yl)amino)-3-((5-isopropyl-2-methoxy-4-methylphenyl)sulfonamido)-4-oxobutanoic Acid

Rink amide resin was used as a solid-phase support (0.05 mmol scale), and standard procedures described in the previous sections were used for the synthesis of compound **21**. The amino acids coupled were Fmoc-d-Trp-OH, Fmoc-Cha-OH, and Fmoc-d-Asp-OH.

### Compound **50**: (3*R*)-4-((2*S*)-2-(((*S*)-1-Amino-3-(1*H*-indol-3-yl)-1-oxopropan-2-yl)carbamoyl)octahydro-1*H*-indol-1-yl)-3-((5-isopropyl-2-methoxy-4-methylphenyl)sulfonamido)-4-oxobutanoic Acid

Rink amide resin was used as a solid-phase support (0.05 mmol scale), and standard procedures described in the previous sections were used for the synthesis of compound **5**. The amino acids coupled were Fmoc-d-Trp-OH, Fmoc-l-octahydroindole-2-carboxylic acid, and Fmoc-d-Asp-OH. Two peaks are observed in the HPLC result both corresponding to the correct mass. Peak 1 (compound **50**) is more active than peak 2 (compound **51**), likely representing two different diastereoisomers (Figures S11 and S12).

### Preparation of the Screening Library and Deconvolution Mixtures

To prepare an isokinetic mixture of Fmoc-amino acids, we considered the mol % of each amino acid or analogues of amino acids.^27,31^ The Fmoc-amino acid mol % values reported in the literature are as follows: Fmoc-Ala-OH, 3.4; Fmoc-Arg(Pbf)-OH, 6.5; Fmoc-Asn(Trt)-OH, 5.3; Fmoc-Asp(*O*-*t*-Bu)-OH, 3.5; Fmoc-Glu(*O*-*t*-Bu)-OH, 3.6; Fmoc-Gln(Trt)-OH, 5.3; Fmoc-Gly-OH, 2.9; Fmoc-His(Trt)-OH, 3.5; Fmoc-Ile-OH, 17.4; Fmoc-Leu-OH, 4.9; Fmoc-Lys(Boc)-OH, 6.2; Fmoc-Nle-OH, 3.8; Fmoc-Phe-OH, 2.5; Fmoc-Pro-OH, 4.3; Fmoc-Ser(*O*-*t*-Bu)-OH, 2.8; Fmoc-Thr(*O*-*t*-Bu)-OH, 4.8; Fmoc-Trp(Boc)-OH, 3.8; Fmoc-Tyr(*O*-*t*-Bu)-OH, 4.1; Fmoc-Val-OH, 11.3.^27^Table S2 reports the amounts used of each amino acid on a 0.1 mmol reaction scale. Each mixture was then preactivated with HATU and Oxyma Pure in DMF (2 mL), and the reaction was agitated for 2 h.

### Protein Expression and Purification

A cDNA fragment encoding the ligand-binding domain of hMcl-1 (residues 172–323) cloned into a pET15b vector with an *N*-terminal His tag was used in the expression of hMcl-1 and methyl-^13^C-l-methionine hMcl-1. The plasmid was transformed into BL21 (DE3) gold pLysS competent cells and grown in LB medium at 37 °C with 100 μg/mL ampicillin until an OD_600_ of 0.6–0.7 was reached followed by induction with 1 mM IPTG overnight at 20 °C. Bacteria were then collected by centrifugation and lysed by sonication. To obtain methyl-^13^C-l-methionine hMcl-1, 100 mg of methyl-^13^C-l-methionine suspended in 1 mL of DMSO per liter of LB medium was added 10 min before induction. The overexpressed proteins containing an N-terminal His tag were purified using immobilized metal ion affinity chromatography (IMAC) with a linear gradient of imidazole (elution buffer: 25 mM Tris at pH 7.5, 500 mM NaCl, and 500 mM imidazole). Finally, the protein was further purified and buffer exchanged, through size-exclusion chromatography with a HiLoad 26/60 Superdex 75 preparative-grade column into an aqueous buffer composed of 50 mM phosphate at pH 7.5, 150 mM NaCl, and 1 mM DTT.

### Nuclear Magnetic Resonance Spectroscopy

NMR spectra were acquired on a Bruker Avance III 700 MHz spectrometer equipped with a TCI cryoprobe. All NMR data were processed and analyzed using TopSpin 4.1.0 (Bruker, Billerica, MA). For the mixture screening, each of the 96 mixtures was dissolved into a 5 mm NMR tube to a final concentration of 2 mM in the presence of 20 μM hMcl-1 in a buffer containing 40 mM Tris pH = 7.5, 150 mM NaCl, and 1 mM DTT. For each mixture, 1D ^1^H-aliph experiments were acquired. For ranking purposes, a total chemical shift perturbation generated by each mixture to the five peaks around 0 ppm in the 1D ^1^H-aliph spectra of hMcl-1 was considered (Figure 4). 2D-[^13^C,^1^H]-HSQC experiments were acquired with 20 μM protein using 32 scans with 2048 and 256 complex data points in the ^1^H and ^13^C dimensions, respectively, at 298 K.

### Isothermal Titration Calorimetry Measurements

Isothermal titration calorimetry measurements were performed using an Affinity ITC autosampler from TA Instruments (New Castle, DE). The titrations were performed in a direct fashion by titrating the ligand solution into the protein. All the measurements were performed at 25 °C dissolving the agents in 50 mM phosphate at pH 7.5, 150 mM NaCl, and a final DMSO concentration of 1%. To test compound **12**, the syringe was filled with a 2 mM solution of compound **12**, and 20 injections of 5 μL each were performed into the cell containing a 100 μM solution of the protein. The injections were made at 200 s intervals with a stirring speed of 75 rpm. To test compound **50**, the syringe was filled with a 500 μM solution of compound **50**, and 20 injections of 5 μL each were performed into the cell containing a 50 μM solution of the protein. The injections were made at 200 s intervals with a stirring speed of 75 rpm. All the solutions were kept in the autosampler at 4 °C. The analysis of the thermodynamics signatures and for dissociation constant determination was performed by NanoAnalyze software (TA Instruments, New Castle, DE) and subsequently exported into Microsoft Excel.

### Molecular Modeling

Docking of compound **50** was accomplished with the Surflex 2.7’s molecular docking module (surflex-dock; Sybyl, Certara) and the PDB ID 6P3P, which is based on conformation optimization procedures implemented for morphological similarity, that fragments the molecule, docks the fragments, and reconstructs the molecule in the active site of the protein. The ligand in 6P3P was used to define a binding region and as a test ligand for the docking protocol. The binding pose predicted by the protocol closely resembled the experimental docked geometry of the experimentally derived binding pose for the ligand (not shown). Subsequently, the molecular model of compound **50** was generated, energy minimized, and docked using the same protocol. Molecular models were analyzed using MOE 2019.0101 (Chemical Computing Group) or Chimera (http://www.cgl.ucsf.edu/chimera).

### DELFIA (Dissociation-Enhanced Lanthanide Fluorescent Immunoassay)

Each well of 96-well streptavidin-coated plates (PerkinElmer) was incubated with 100 μL of a 600 ng/mL biotinylated-BH3 peptide of sequence biotin-amino-hexanoic acid-EDIIRNIARHLAQVGDSMDR-NH_2_ for 2 h and washed 3 times with a wash solution (PerkinElmer). Subsequently, a mixture containing 11 μL of protein and a serial dilution of the test compounds as well as 89 μL of an Eu-N1-labeled anti-6x-His antibody (PerkinElmer) was added to each well and incubated for 2 h. Plates were then washed 3 times, and 200 μL of enhancement solution (PerkinElmer) was added to each well. After a 10 min incubation, fluorescence measurements were taken with a Victor X5 microplate reader with the excitation and emission wavelengths of 340 and 615, respectively. All the incubations were performed at room temperature. The final protein concentrations used for hBfl-1 and hMcl-1 were 15 and 16 nM, respectively and the final anti-6x-His antibody used was 22.2 ng/well. Fluorescence readings were normalized to those of 1% DMSO-treated wells and reported as % inhibition. Prism 9 (GraphPad) was used to calculate IC_50_ values.

## Supporting Information Available

Molecular formula String file with key compounds (CSV)
Atomic coordinates of the model between hMcl-1 and compound **50** (PDB)
(Table S1) Structures of the 96 sulfonyl chlorides present in P1 in the library, (Table S2) molar equivalents of each amino acid used for the preparation of mixtures, (Table S3) analogues of compound **12**, (Table S4) SAR data on compound **21**, (Table S5) mass spectrometry data of compounds, (Figure S1) deconvolution approach used to identify P2 and P3, (Figure S2) experimental data of the second step of the deconvolution approach, (Figure S3) experimental data of the third step of the deconvolution approach: mixtures in P2, (Figure S4) experimental data of the deconvolution approach applied to a negative mixture, (Figure S5) experimental data relative to the elongation of the agents in position 4, (Figure S6) experimental data of the identification of d-Trp as the best amino acid in P4, (Figure S7) biophysical characterization of compound **21** binding to hMcl-1, (Figure S8) ITC curves of compounds **59** and **60** against hMcl-1, (Figure S9) 2D [^1^H,^13^C] correlation spectrum of 20 μM ^13^C^ε^-Met hMcl-1 in the presence of different concentrations of compound **50**, (Figure S10) HPLC trace for compound **21**, (Figure S11) HPLC trace for compound **50** (peak 1), and (Figure S12) HPLC trace for compound **51** (peak 2) (PDF)

## Supplementary Material

jm3c01073_si_001.csv

jm3c01073_si_002.pdb

## Abbreviations

DELFIA: dissociation-enhanced lanthanide fluorescent immunoassay
DMF: dimethylformamide
HATU: 1-[bis(dimethylamino)methylene]-1*H*-1,2,3-triazolo[4,5-*b*]pyridinium 3-oxid hexafluorophosphate
DIPEA: *N*,*N*-diisopropylethylamine
DBU: 1,8-diazabicyclo[5.4.0]undec-7-ene
DTT: dithiothreitol
OXYMA: ethyl cyanoglyoxylate-2-oxime
THF: tetrahydrofuran
