# Supplementary material for: Mixture-Based Screening of Focused Combinatorial Libraries by NMR: Application to the Antiapoptotic Protein hMcl-1
Source: J Med Chem. 2023 Jul 19;66(14):10108–18. doi: 10.1021/acs.jmedchem.3c01073 (PMC10388297; doi:10.1021/acs.jmedchem.3c01073)
Supplement: Supplementary file 3 — jm3c01073_si_003.pdf [file jm3c01073_si_003.pdf]

Supporting Information

Mixture-based screening of focused combinatorial libraries by NMR: application to the anti-apoptotic protein hMcl-1

Giulia Alboreggia,[1] Parima Udompholkul[1], Carlo Baggio[1], and Maurizio Pellecchia[1]*

[1]*Division of Biomedical Sciences, School of Medicine, University of California Riverside, 900 University Avenue, Riverside, CA 92521, USA.*

*Corresponding author: Maurizio Pellecchia, phone number: (951) 827-7829; email address: maurizio.pellecchia@ucr.edu

Table of Contents:

Table S1 page S3
Structures of the 96 sulfonyl chlorides present in P1 in the library.
Table S2 page S12
Mol% of each amino acid used for the preparation of mixtures and mmol used, considering a 0.1 mmol scale.
Table S3 page S14
Analogues of Compound (**12**): SAR in P2, and relative CSP in the 1D-^{1}H-*aliphatic* spectrum.
Table S4 page S16
SAR of Compound (**20**), and relative CSP in the 1D-^{1}H-*aliphatic* spectrum.
Table S5 page S22
Mass-spectrometry data of compounds. All the compounds were analyzed using an Agilent 6545 QTOF LC/MS instrument.
Figure S1 page S24
Deconvolution approach used to identify P2 and P3.
Figure S2 page S25
Experimental data of the second step of the deconvolution approach.
Figure S3 page S26
Experimental data of the third step of the deconvolution approach: mixtures in P2.
Figure S4 page S27
Experimental data of the deconvolution approach for the negative mixture D05.
Figure S5 page S28
Experimental data of the addition of another amino acid in position 4 (P4) using the same deconvolution approach.
Figure S6 page S29
Experimental data of the identification of D-Trp as the best amino acid in P4.
Figure S7 page S30
Biophysical characterization of Compound (**21**) binding to hMcl-1.

Figure S8 page S31
ITC curves of hMcl-1 in the presence of Compound (**59**) and Compound (**60**).
Figure S9 page S32
2D [^{1}H,^{13}C] correlation spectrum of 20 μM ^{13}C-e-Met hMcl-1 in presence of different concentration of Compound (**50**).
Figure S10 page S33
HPLC trace for Compound (**21**).
Figure S11 page S34
HPLC trace for Compound (**50**) (peak 1).
Figure S12 page S35
HPLC trace for Compound (**51**) (peak 2).

Table S1. Structures of the 96 sulfonyl chlorides present in P1 in the library.

**Mol Name**	**Structure**
4-(aminocarbonyl)benzenesulfonyl chloride	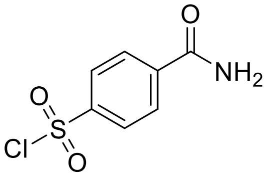
3-chloro-4-methoxybenzenesulfonyl chloride	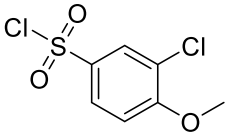
4-methoxy-3,5-dimethylbenzenesulfonyl chloride	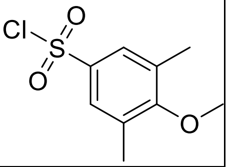
1,5-dimethyl-1H-pyrazole-4-sulfonyl chloride	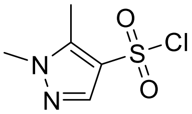
1,3-dimethyl-2,4-dioxo-1,2,3,4-tetrahydro-6-quinazolinesulfonyl chloride	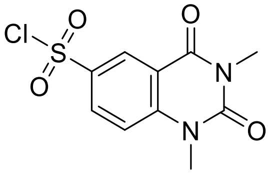
sulfamoyl chloride	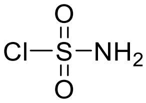
(4-chloro-2-fluorophenyl)methanesulfonyl chloride	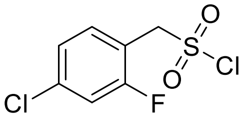
4-isobutylbenzenesulfonyl chloride	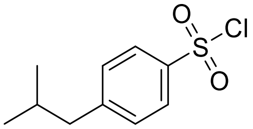
2,4,5-trimethylbenzenesulfonyl chloride	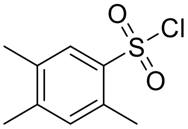
3,4-dimethylbenzenesulfonyl chloride	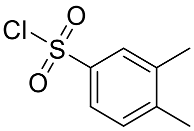

4-(2-oxopyrrolidin-1-yl)benzenesulfonyl chloride	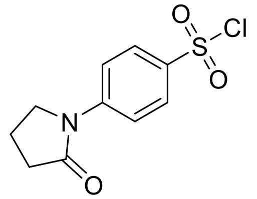
3-chloro-2-methylbenzenesulfonyl chloride	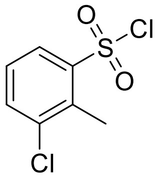
4-isopropylbenzenesulfonyl chloride	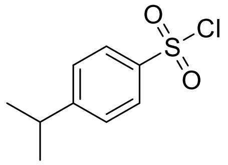
2,5-dimethoxybenzenesulfonyl chloride	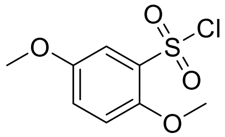
3,5-difluorobenzenesulfonyl chloride	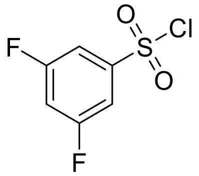
5-chlorothiophene-2-sulfonyl chloride	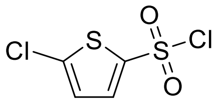
3-chloro-4-fluorobenzenesulfonyl chloride	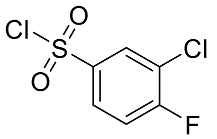
4-propylbenzenesulfonyl chloride	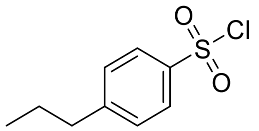
(4-chlorophenyl)methanesulfonyl chloride	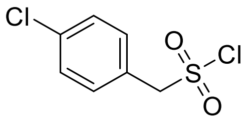
3-methyl-2-oxo-2,3-dihydro-1,3-benzoxazole-6-sulfonyl chloride	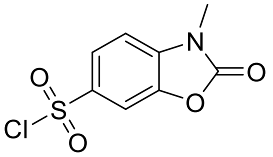
3,5-dimethyl-1H-pyrazole-4-sulfonyl chloride	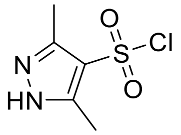

2,4-dimethoxybenzenesulfonyl chloride	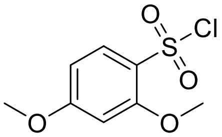
3-pyridinesulfonyl chloride hydrochloride	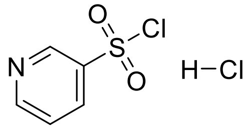
(3-methylphenyl)methanesulfonyl chloride	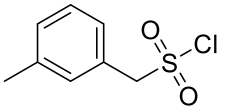
(2-chlorophenyl)methanesulfonyl chloride	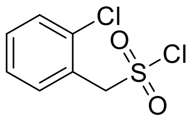
(2-methylphenyl)methanesulfonyl chloride	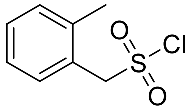
4-ethoxybenzenesulfonyl chloride	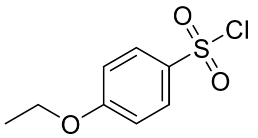
3-oxo-3,4-dihydro-2H-1,4-benzoxazine-6-sulfonyl chloride	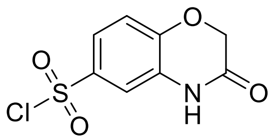
3-fluoro-4-methoxybenzenesulfonyl chloride	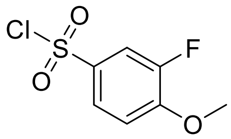
4-methoxy-3-methylbenzenesulfonyl chloride	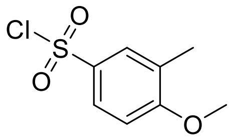
4-methoxy-2,5-dimethylbenzenesulfonyl chloride	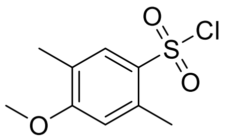
(4-fluorophenyl)methanesulfonyl chloride	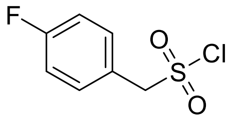
5-(5-isoxazolyl)-2-thiophenesulfonyl chloride	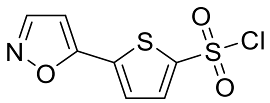

4-(2-amino-2-oxoethoxy)benzenesulfonyl chloride	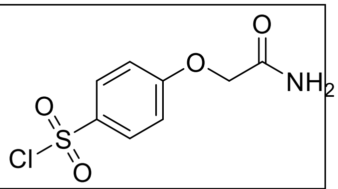
4-ethoxy-3-methylbenzenesulfonyl chloride	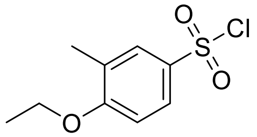
3,4-dihydro-2H-1,5-benzodioxepine-7-sulfonyl chloride	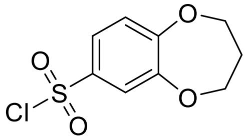
2-(trifluoroacetyl)-1,2,3,4-tetrahydro-7-isoquinolinesulfonyl chloride	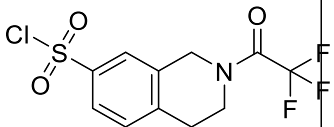
3-[(dimethylamino)carbonyl]-4-methoxybenzenesulfonyl chloride	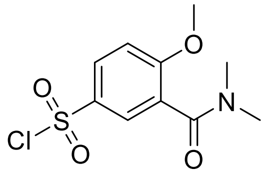
1-ethyl-5-methyl-1H-pyrazole-4-sulfonyl chloride	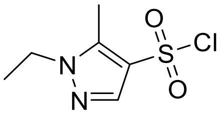
1-methyl-1H-pyrazole-4-sulfonyl chloride	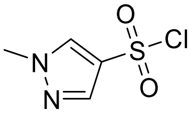
3-methyl-2,4-dioxo-1,2,3,4-tetrahydro-6-quinazolinesulfonyl chloride	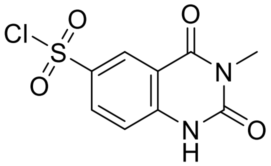
1-acetyl-1,2,3,4-tetrahydro-6-quinolinesulfonyl chloride	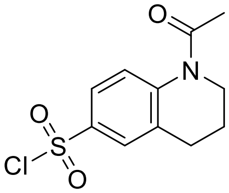
4-(acetylamino)-1-naphthalenesulfonyl chloride	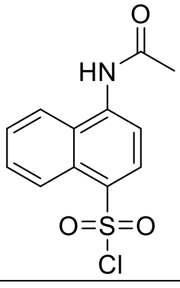

2,4-dichloro-3-methylbenzenesulfonyl chloride	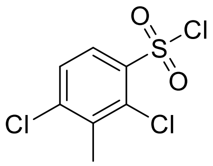
5-tert-butyl-2,3-dimethylbenzenesulfonyl chloride	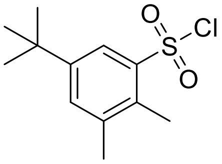
5,7-dimethyl-2,1,3-benzoxadiazole-4-sulfonyl chloride	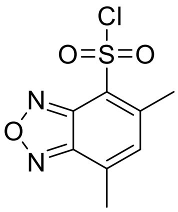
5-(acetylamino)-2-methoxybenzenesulfonyl chloride	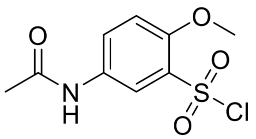
2-methoxy-4,5-dimethylbenzenesulfonyl chloride	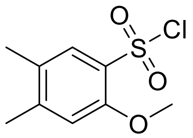
4-ethoxy-1-naphthalenesulfonyl chloride	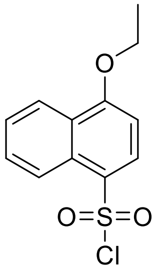
4-methoxy-5,6,7,8-tetrahydro-1-naphthalenesulfonyl chloride	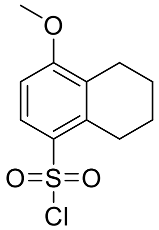
2-oxo-1,2-dihydrobenzo[cd]indole-6-sulfonyl chloride	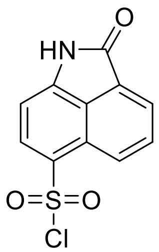

4-methoxy-1-naphthalenesulfonyl chloride	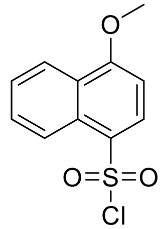
5-methoxy-2,4-dimethylbenzenesulfonyl chloride	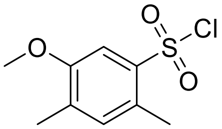
2,5-dimethoxy-4-methylbenzenesulfonyl chloride	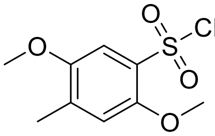
5-isopropyl-2-methoxy-4-methylbenzenesulfonyl chloride	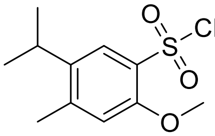
5-isopropyl-2-methoxybenzenesulfonyl chloride	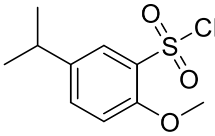
2-phenoxyethanesulfonyl chloride	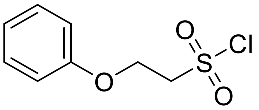
2-(2-fluorophenoxy)ethanesulfonyl chloride	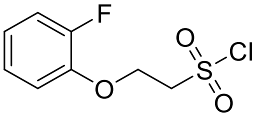
5-chloro-2-ethoxy-4-methylbenzenesulfonyl chloride	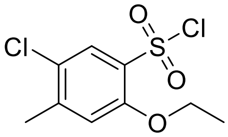
2-(4-methylphenoxy)ethanesulfonyl chloride	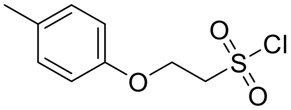
2-ethoxy-5-methylbenzenesulfonyl chloride	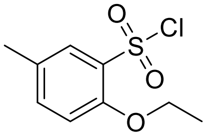
4,5-dichloro-2-methylbenzenesulfonyl chloride	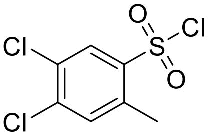
5-[5-(trifluoromethyl)-3-isoxazolyl]-2-furansulfonyl chloride	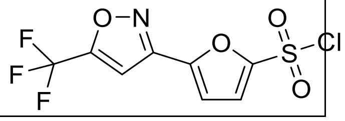

2-methyl-5-[5-(trifluoromethyl)-3-isoxazolyl]benzenesulfonyl chloride	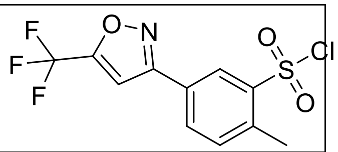
(2,4-difluorophenyl)methanesulfonyl chloride	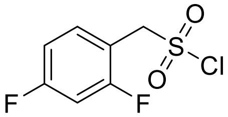
5-(5-isoxazolyl)-2-methoxybenzenesulfonyl chloride	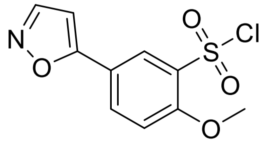
5-(5-isoxazolyl)-2-methyl-3-thiophenesulfonyl chloride	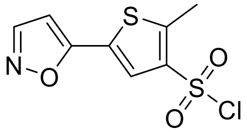
3-isopropyl-4-methoxybenzenesulfonyl chloride	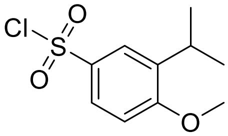
2-methyl-5-[5-(trifluoromethyl)-3-isoxazolyl]-3-thiophenesulfonyl chloride	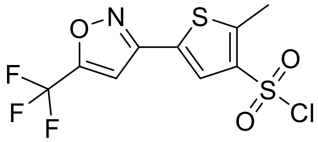
2-methyl-5-(2-methyl-1,3-oxazol-5-yl)benzenesulfonyl chloride	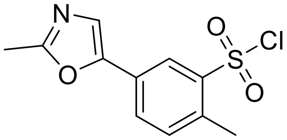
4-[5-(trifluoromethyl)-3-isoxazolyl]-2-thiophenesulfonyl chloride	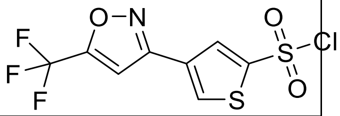
2-methyl-5-[5-(trifluoromethyl)-3-isoxazolyl]-3-furansulfonyl chloride	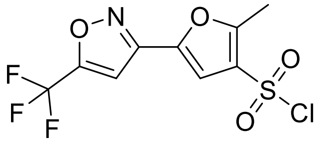
1-isopropyl-3,5-dimethyl-1H-pyrazole-4-sulfonyl chloride	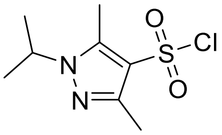
5-(3,4-dimethyl-5-isoxazolyl)-2-furansulfonyl chloride	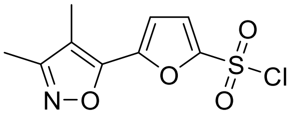
2,5-dichloro-4-methoxybenzenesulfonyl chloride	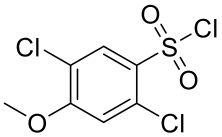

2-methyl-5-(3-methyl-5-isoxazolyl)-3-thiophenesulfonyl chloride	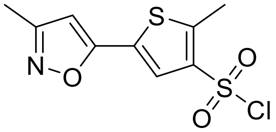
2-methyl-5-(1H-pyrazol-1-yl)benzenesulfonyl chloride	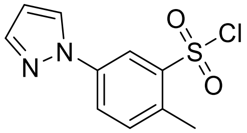
2-methoxy-5-(1H-pyrazol-1-yl)benzenesulfonyl chloride	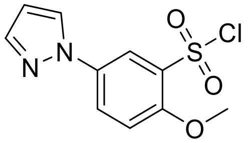
4-methoxy-3-[(methylamino)carbonyl]benzenesulfonyl chloride	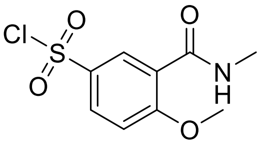
5-(3,5-dimethyl-4-isoxazolyl)-2-thiophenesulfonyl chloride	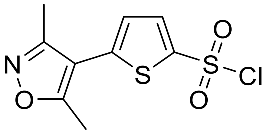
2-methyl-8-quinolinesulfonyl chloride	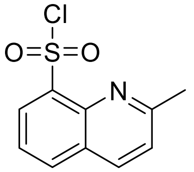
2-chloro-4-fluoro-5-methylbenzenesulfonyl chloride	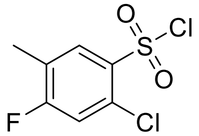
4-(aminocarbonyl)-2-thiophenesulfonyl chloride	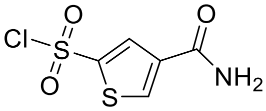
2-methoxybenzenesulfonyl chloride	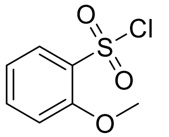
2-ethoxybenzenesulfonyl chloride	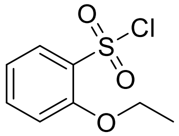
2,3-dihydro-1,4-benzodioxine-6-sulfonyl chloride	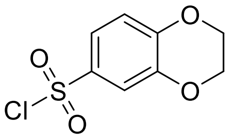

2,3-dihydro-1,4-benzodioxine-5-sulfonyl chloride	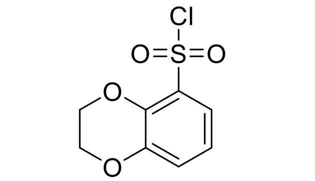
1H-benzimidazole-5-sulfonyl chloride hydrochloride	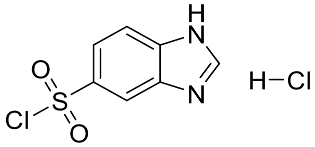
2-oxo-1,2,3,4-tetrahydro-6-quinolinesulfonyl chloride	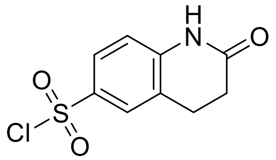
4-(5-isoxazolyl)-2-thiophenesulfonyl chloride	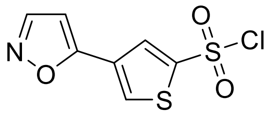
2-oxo-2,3-dihydro-1H-benzimidazole-5-sulfonyl chloride	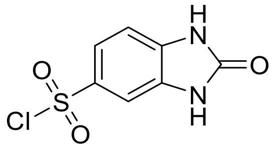
tetrahydro-2H-pyran-4-sulfonyl chloride	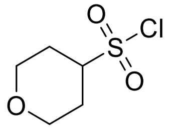
5-(3-methyl-5-isoxazolyl)-2-furansulfonyl chloride	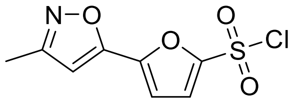
5-methoxy-2,4-dimethylbenzenesulfonyl chloride	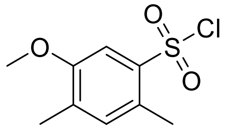
3,5-dimethyl-1-phenyl-1H-pyrazole-4-sulfonyl chloride	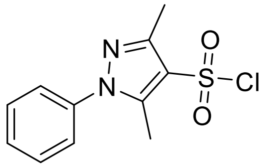
4-(2-methyl-1,3-oxazol-5-yl)benzenesulfonyl chloride	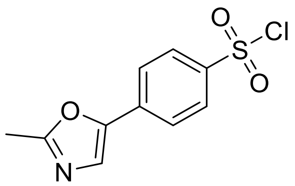

Table S2. Relative equivalents used of each amino acid in the preparation of mixtures. The exact amounts used in our 0.1 mmol scale is also reported.

**Building Block**	**Relative number of equivalents**	**mmol used in the reaction on a 0.1 mmol scale**
**Sub-mixture 1**		
Fmoc-D-Dab(Boc)-OH	1.794	0.054
Fmoc-L-Dab(Boc)-OH	1.794	0.054
Fmoc-D-Dap(Boc)-OH	1.794	0.054
Fmoc-L-Dap(Boc)-OH	1.794	0.054
Fmoc-D-Orn(Boc)-OH	1.794	0.054
Fmoc-L-Orn(Boc)-OH	1.794	0.054
Fmoc-D-Lys(Boc)-OH	1.794	0.054
Fmoc-L-Lys(Boc)-OH	1.794	0.054
Fmoc-L-His(Boc)-OH	1	0.030
Fmoc-D-His(Trt)-OH	1	0.030
**Sub-mixture 2**		
Fmoc-D-Asp(OtBu)-OH	1	0.038
Fmoc-L-Asp(OtBu)-OH	1	0.038
Fmoc-L-Glu(OtBu)-OH	1.029	0.039
Fmoc-D-Glu(OtBu)-OH	1.029	0.039
Fmoc-L-Aad(OtBu)-OH	1.029	0.039
Fmoc-Tyr(PO(Obzl)OH)-OH	1.176	0.044
Fmoc-D-Pro-OH	1.235	0.046
Fmoc-Pro-OH	1.235	0.046
**Sub-mixture 3**		
Fmoc-L-Trp(Boc)-OH	1.542	0.066
Fmoc-D-Trp(Boc)-OH	1.542	0.066
Fmoc-L-4,4'-biphenylalanine	1.25	0.054
Fmoc-L-Tyr(tBu)-OH	1.667	0.071
Fmoc-D-Tyr(tBu)-OH	1.667	0.071
Fmoc-L-Phe(4-F)-OH	1	0.043
Fmoc-3-(3'-pyridyl)-L-alanine	1.250	0.054
**Sub-mixture 4**		
Fmoc-L-Ala-OH	1	0.027
Fmoc-D-Ala-OH	1	0.027
Fmoc-cycloleucine	3.364	0.092
1-(Fmoc-amino)cyclohexanecarboxylic acid	3.364	0.092
Fmoc-L-Cha-OH	3.364	0.092
Fmoc-D-Cha-OH	3.364	0.092
Fmoc-L-Chg-OH	3.364	0.092
Fmoc-D-Chg-OH	3.364	0.092
Fmoc-L-Nle-OH	1.121	0.031

Fmoc-D-Nle-OH	1.121	0.031
Fmoc-Aib-OH	1	0.027

Table S3. Analogues of tri-peptoide Compound (**12**): SAR in P2. CSP 2 represents the shift of the peak at -0.08 ppm. CSP1 represents the total shift of the two peaks overlapping at -0.616 ppm, which shift in different directions in presence of a compound. +: 0.01 ppm < CSP < 0.05 ppm; ++: 0.05 ppm < CSP < 0.1 ppm; +++: 0.1 ppm < CSP < 0.13 ppm; ++++: 0.13 ppm < CSP < 0.15 ppm; +++++: CSP > 0.15 ppm. N/A means that no shift has been detected.

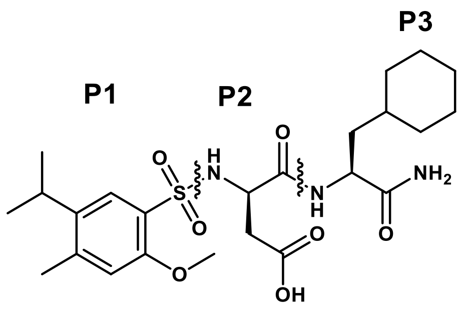<br>Compound (**12**)		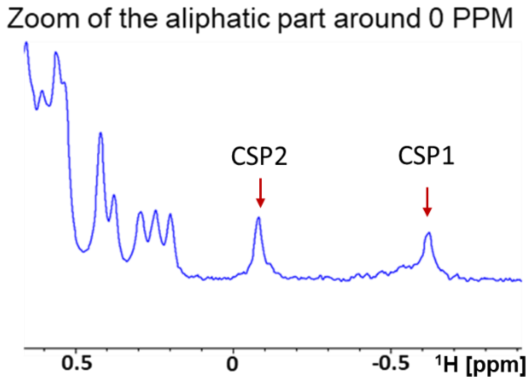	
**Analogues of D-Asp in P2**	**Name**	**CSP2**	**CSP1**
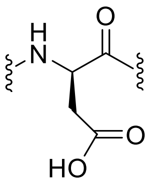	Compound (**12**)	**N/A**	++
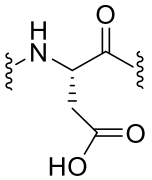	Compound (**13**)	**N/A**	+
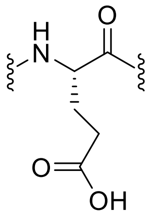	Compound (**14**)	**N/A**	+
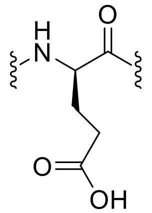	Compound (**15**)	**N/A**	+

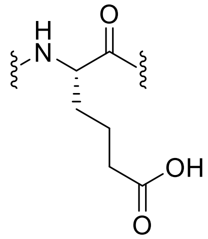	Compound (**16**)	**N/A**	+
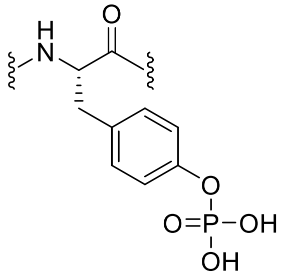	Compound (**17**)	**N/A**	++

Table S4. SAR on Compound (**20**)**.** CSP 2 represents the shift of the peak at -0.08 ppm. CSP1 represents the total shift of the two peaks overlapping at -0.616 ppm, which shifts in different directions in presence of a compound. +: 0.01 ppm < CSP < 0.05 ppm; ++: 0.05 ppm < CSP < 0.1 ppm; +++: 0.1 ppm < CSP < 0.13 ppm; ++++: 0.13 ppm < CSP < 0.15 ppm; +++++: CSP > 0.15 ppm. N/A means that no shift has been detected.

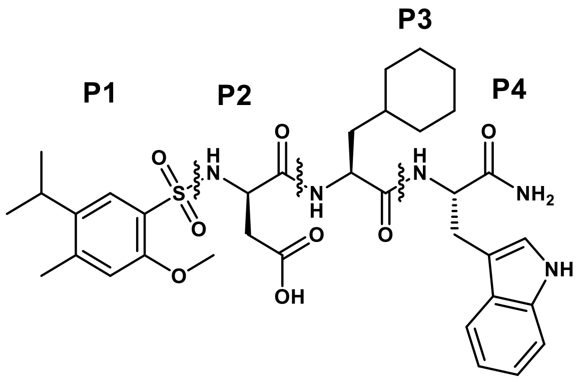

Compound (**20**)

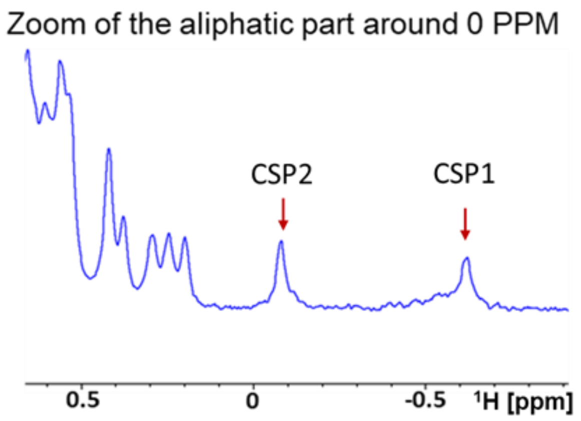

Analogues of L-Trp in P4	Name	CSP2	CSP1
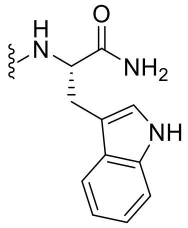	Compound (**20**)	**N/A**	+++
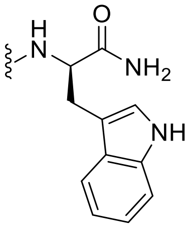	Compound (**21**)	**N/A**	+++
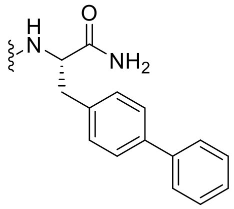	Compound (**22**)	**N/A**	++

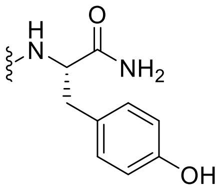	Compound (**23**)	**N/A**	++
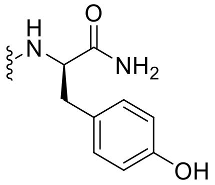	Compound (**24**)	**N/A**	++
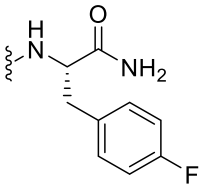	Compound (**25**)	**N/A**	+++
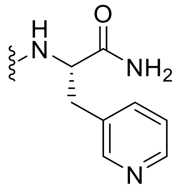	Compound (**26**)	**N/A**	++
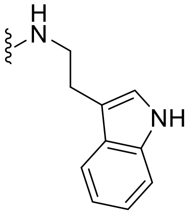	Compound (**27**)	**N/A**	+++
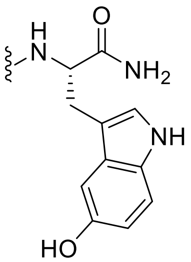	Compound (**28**)	**N/A**	++
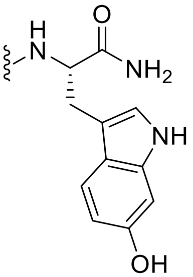	Compound (**29**)	**N/A**	++

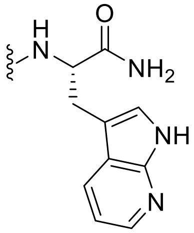	Compound (**30**)	**N/A**	+++
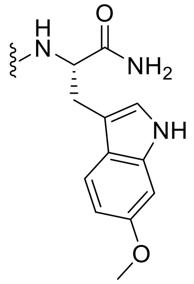	Compound (**31**)	**N/A**	++
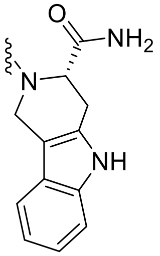	Compound (**32**)	**N/A**	+++
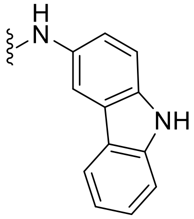	Compound (**33**)	**N/A**	++
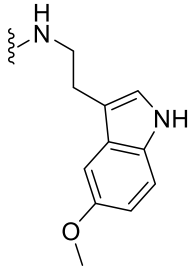	Compound (**40**)	**N/A**	+++
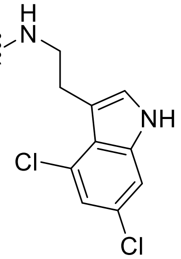	Compound (**41**)	**N/A**	++++

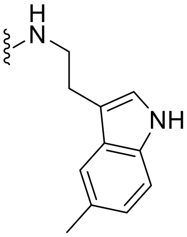	Compound (**42**)	**N/A**	+++
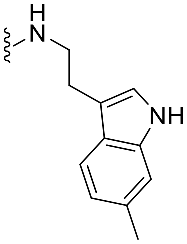	Compound (**43**)	**N/A**	++++
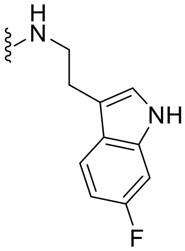	Compound (**44**)	**N/A**	++++
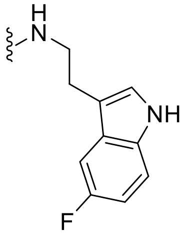	Compound (**45**)	**N/A**	++++
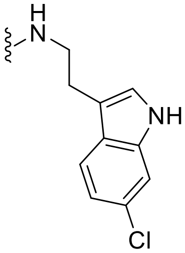	Compound (**48**)	**N/A**	++++
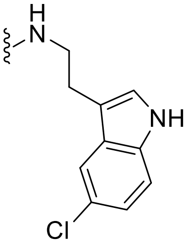	Compound (**49**)	**N/A**	+++++
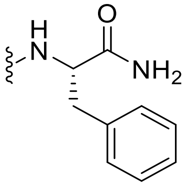	Compound (**57**)	**N/A**	+++

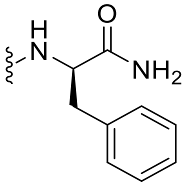	Compound (**58**)	**N/A**	++
**Analogues of L-Cha in P3**			
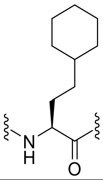	Compound (**37**)	**N/A**	++++
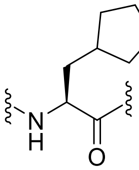	Compound (**38**)	**N/A**	++++
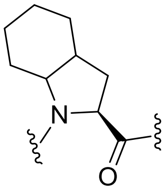	Compound (**50**)	+	+++
**Analogues of E07 sulfonyl chloride in P1**			
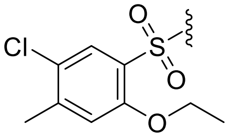	Compound (**34**)	**N/A**	+++
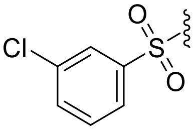	Compound (**35**)	**N/A**	++
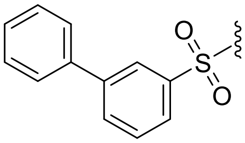	Compound (**36**)	**N/A**	+++
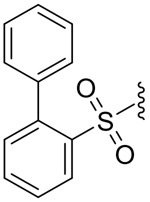	Compound (**39**)	**N/A**	+++++

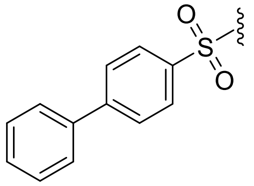	Compound (**46**)	**N/A**	+++
**Combination**			
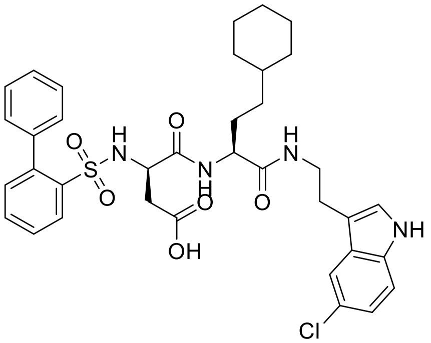	Compound (**49**)	**N/A**	+++
**Addition of P5**			
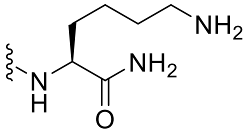	Compound (**52**)	**N/A**	+++
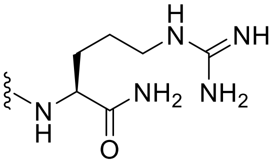	Compound (**53**)	**N/A**	+++
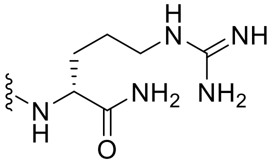	Compound (**54**)	**N/A**	+++
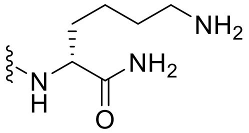	Compound (**55**)	**N/A**	+++
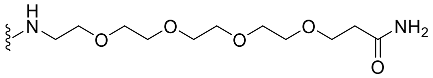	Compound (**56**)	**N/A**	+++

Table S5. Mass-spectrometry data of the compounds. All the compounds were analyzed using an Agilent 6545 QTOF LC/MS instrument.

**ID**	**Calcd [M]**	**Obs. $[M+H]^+$ (m/z)**
Compound (**1**)	385.1682	386.1753
Compound (**2**)	427.2151	428.2222
Compound (**3**)	399.1840	400.1909
Compound (**4**)	385.1696	386.1752
Compound (**5**)	425.2011	426.2066
Compound (**6**)	439.2167	440.2221
Compound (**7**)	467.2470	468.2544
Compound (**8**)	467.2469	468.2542
Compound (**9**)	453.2309	454.2383
Compound (**10**)	453.2316	454.2390
Compound (**11**)	427.2157	428.2231
Compound (**12**)	511.2359	512.2430
Compound (**13**)	511.2360	512.2431
Compound (**14**)	525.2515	526.2582
Compound (**15**)	525.2519	526.2581
Compound (**16**)	539.2673	540.2743
Compound (**17**)	639.2365	640.2446
Compound (**18**)	493.2627	494.2700
Compound (**19**)	493.2630	494.2697
Compound (**20**)	697.3164	698.3221
Compound (**21**)	697.3163	**$[M+Na]^+$** = 720.3057
Compound (**22**)	734.3392	735.3468
Compound (**23**)	674.3029	675.3101
Compound (**24**)	674.3031	675.3092
Compound (**25**)	676.2932	677.2994
Compound (**26**)	659.2998	660.3074
Compound (**27**)	654.3083	655.3152
Compound (**28**)	713.3080	714.3155
Compound (**29**)	713.3094	714.3160
Compound (**30**)	698.3103	699.3179
Compound (**31**)	727.3225	728.3284

Compound (**32**)	709.3128	**[M+Na]$^+$ =** 732.3027
Compound (**33**)	676.2902	677.2975
Compound (**34**)	703.2416	704.2494
Compound (**35**)	645.1999	646.2054
Compound (**36**)	687.2735	688.2803
Compound (**37**)	711.3272	712.3344
Compound (**38**)	683.3019	684.3097
Compound (**39**)	687.2732	688.2804
Compound (**40**)	684.3179	685.3255
Compound (**41**)	722.2295	723.2370
Compound (**42**)	668.3242	669.3310
Compound (**43**)	668.3234	669.3301
Compound (**44**)	672.2990	673.3059
Compound (**45**)	672.2999	673.3071
Compound (**46**)	687.2716	688.2793
Compound (**47**)	688.2674	689.2749
Compound (**48**)	688.2695	689.2770
Compound (**49**)	692.2420	693.2499
Compound (**50**)	695.2982	696.3052
Compound (**51**)	695.2981	696.3056
Compound (**52**)	825.4095	826.4146
Compound (**53**)	853.4178	854.4254
Compound (**54**)	853.4187	854.4262
Compound (**55**)	825.4127	826.4203
Compound (**56**)	944.4593	945.4670
Compound (**57**)	658.3047	659.3123
Compound (**58**)	658.3032	659.3108
Compound (**59**)	725.3115	726.3191
Compound (**60**)	681.2832	682.2904

Figure S1. Schematic representation of the deconvolution approach used for the HIT identification: one position at a time, P3, as in this case, or P2, is deconvoluted in order to find the best amino acid that fits that position. In the first step of the deconvolution approach, 4 sub-mixtures are synthesized with the sulfonamide in P1, Ala in P2 and a mixture of nine amino acids in P3 (S1 A). Those four sub-mixtures are analyzed by NMR and the one that creates the bigger CSP in the 1D-^{1}H-*aliphatic* spectrum is further deconvoluted through the synthesis of individual compounds (S1 B). The individual compounds are then tested by NMR to identify the best amino acid that fits P3; the same deconvolution approach is then repeated to study P2, fixing the sulfonamide in P1 and the amino acid found previously in P3.

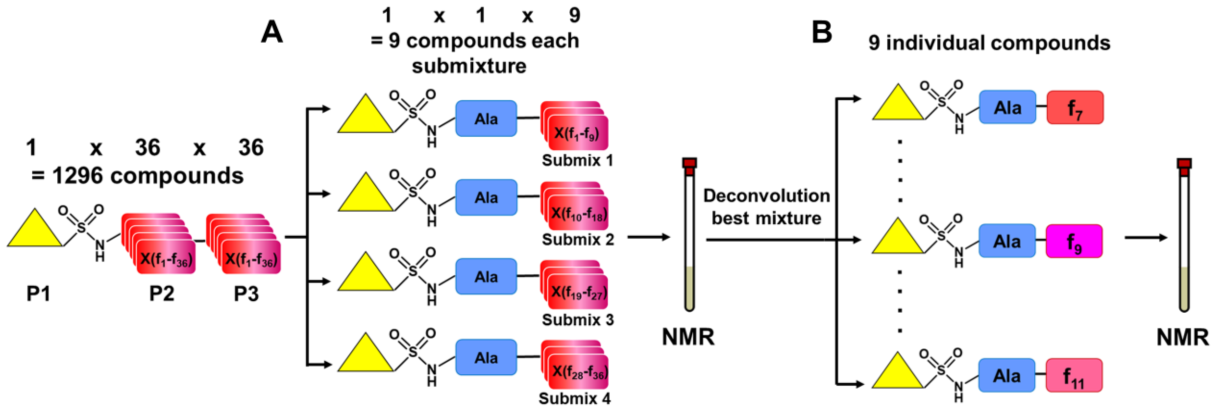

Figure S2. Experimental data of the second step of the deconvolution approach. A) Structures of the compounds synthesized with E07 sulfonamide in P1, Ala in P2 and individual amino acids of the sub-mixture 4 in P3. B) Analysis by NMR of 20μM hMcl-1 alone (blue) and in presence of each individual compound (250μM). Compound (**7**) is the compound that presents the bigger CSP in the 1D-^{1}H-*aliphatic* region of hMcl-1.

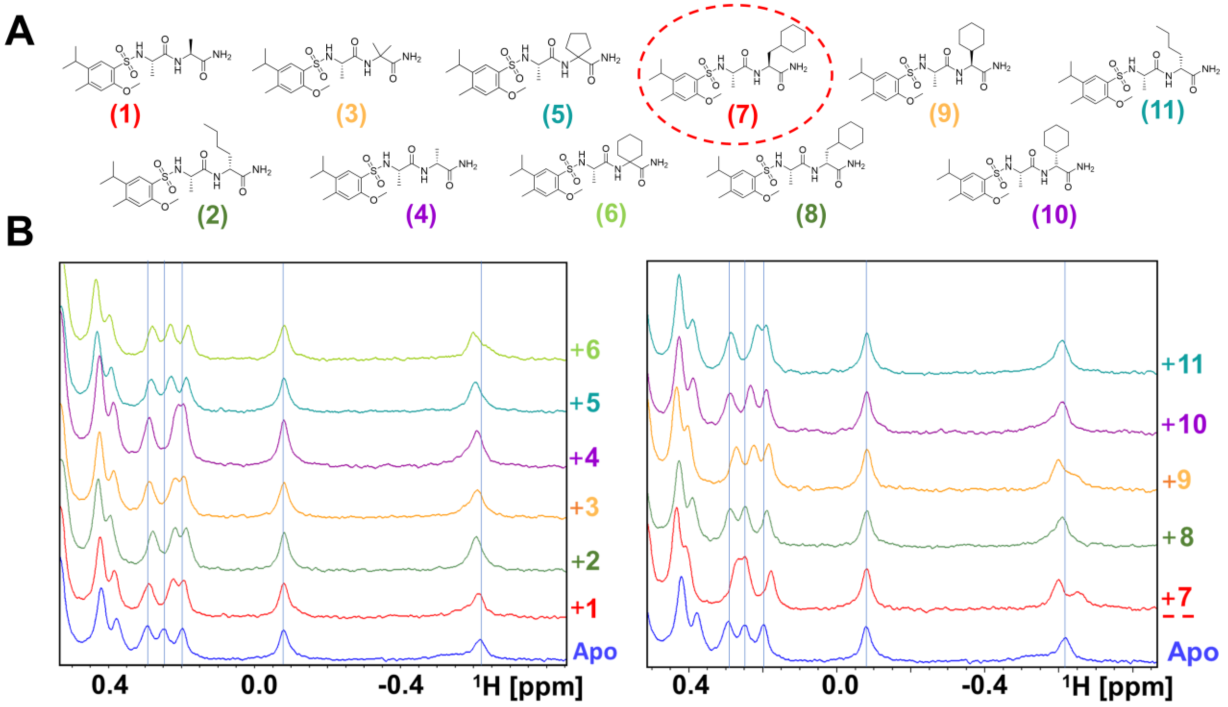

Figure S3. Experimental data of the third step of the deconvolution approach: study of P2. A) Structures of the four sub-mixtures synthesized with E07 sulfonamide in P1, sub-mixtures of amino acids in P2 and Cha in P3. B) Analysis of the four sub-mixtures by NMR: representation of the CSP that they cause in the 1D-^{1}H-*aliphatic* region of the hMcl-1 spectrum.

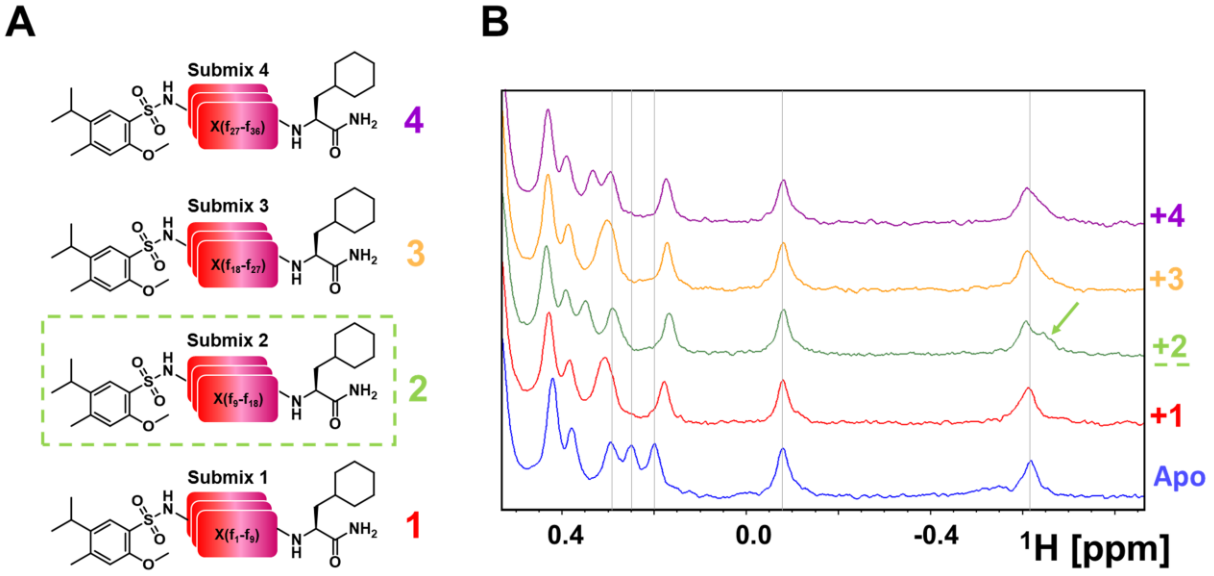

Figure S4. A) Aliphatic region of the 1D ^{1}H NMR spectra of hMcl-1 (20 μM) recorded in the absence (blue) and presence (red) of mixture D05 (2 mM). B) 1D ^{1}H NMR spectra of the sub-mixtures synthesized with D05 sulfonamide in P1, Ala in P2 and four smaller submixtures in P3. C) 1D ^{1}H NMR spectra of the sub-mixtures synthesized with D05 sulfonamide in P1, four smaller submixtures in P2 and Ala in P3.

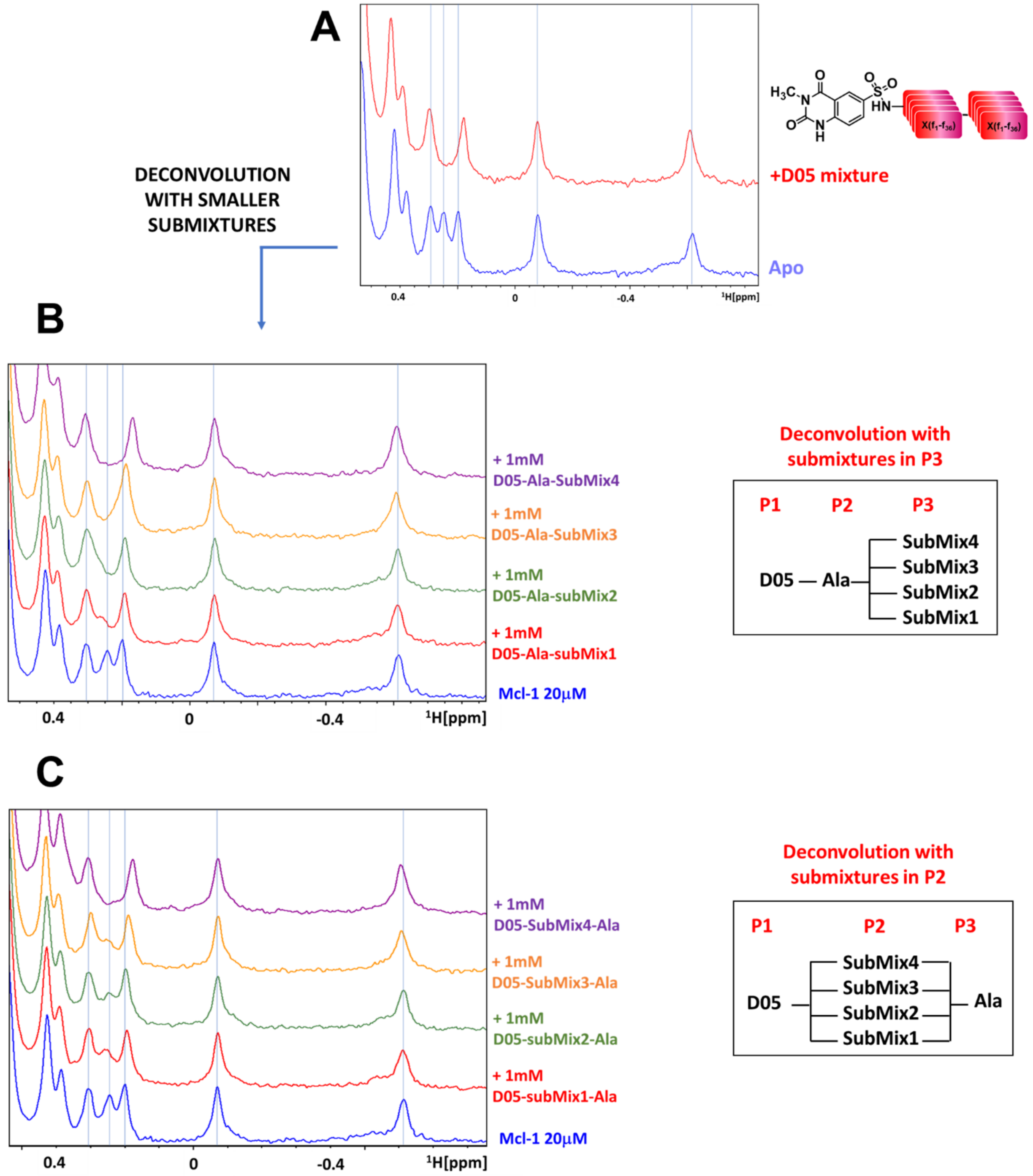

Figure S5. Experimental data of the addition of another amino acid in position 4 (P4) using the same deconvolution approach described before. A) Structures of the four sub-mixtures synthesized with E07 sulfonamide in P1, D-Asp in P2, Cha in P3 and mixtures of amino acids in P4. B) Analysis by NMR of 20μM hMcl-1 alone (blue) and in presence of each mixture (500μM). Mixture 3 is the one that presents the bigger CSP in the 1D-^{1}H-*aliphatic* region of hMcl-1.

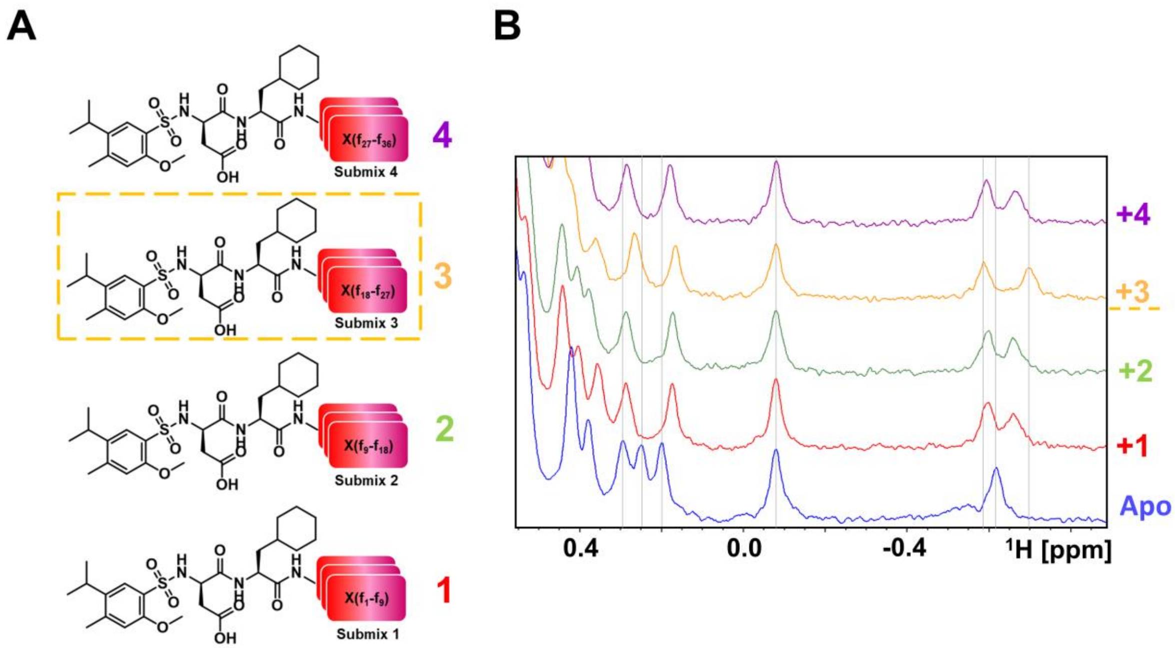

Figure S6. Experimental data of the identification of D-Trp as the best amino acid in P4 using the deconvolution approach. A) Structures of the compounds synthesized with E07 sulfonamide in P1, D-Asp in P2, Cha in P3 and individual amino acids of the sub-mixture 3 in P4. B) Analysis by NMR of 20μM hMcl-1 alone (blue) and in presence of each individual compound at 100μM. Compound (**21**) is the compound that presents the bigger CSP in the 1D-^{1}H-*aliphatic* region of hMcl-1.

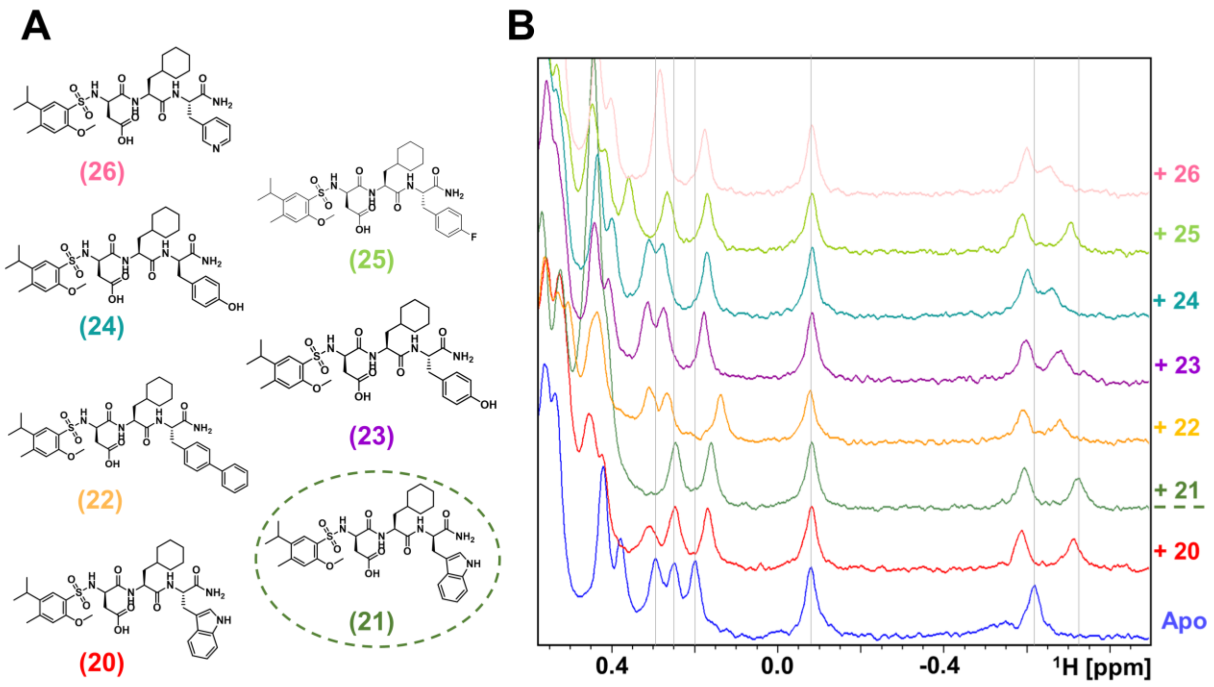

Figure S7. Biophysical characterization of Compound (**21**) binding to hMcl-1. A) Chemical structure of agent Compound (**21**). B) Non-linear fitting K_d estimation following the shift of peak 1 (indicated with an * in panel C) at different concentration points. C) Titration by NMR of Compound (**21**) binding to hMcl-1. D) Perturbations induced by Compound (**21**) (at 100 μM) in the 1D-^{1}H-*aliphatic* region of hMcl-1(20 μM), compared to the perturbations of Compound (**12**) at 250μM (green) and of 2mM E07 mixture (red).

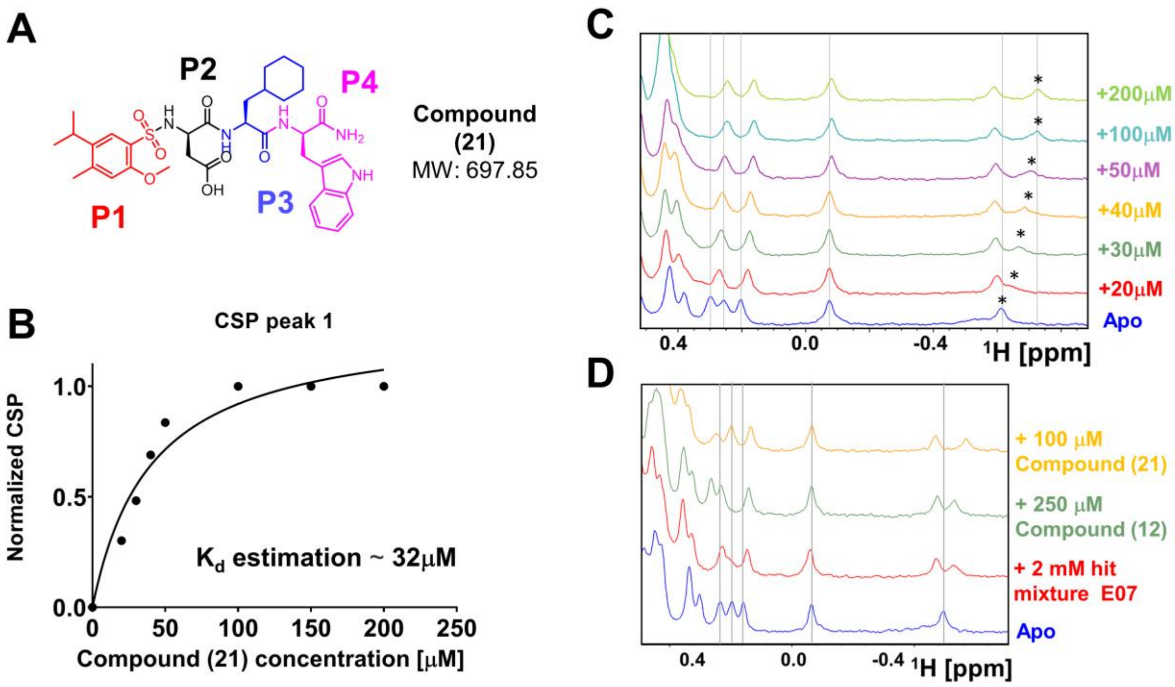

Figure S8. Isothermal titration calorimetry experiments of Compound (**59**) (A) and Compound (**60**) (B) against hMcl-1. Observed K_d values are in the low micromolar range: K_d (Compound (**59**)) = 4.62 ± 0.37 μM (n=2) and K_d (Compound (**60**)) = 2.84 ± 0.67 μM (n=2).

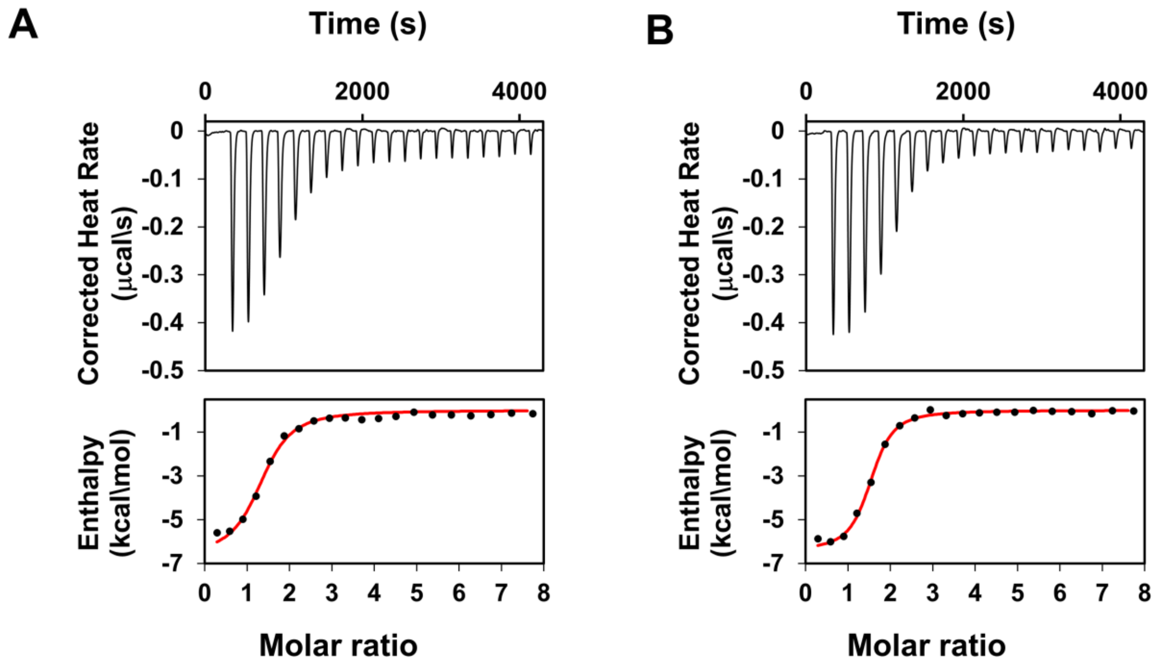

Figure S9. 2D [^{1}H,^{13}C] correlation spectrum of 20 µM ^{13}C-ε-Met hMcl-1 (blue) in presence of 10µM (yellow), 25µM (green) and 100µM (red) of Compound (**50**). The red arrows show the hypothesized shift of the crosspeaks that characterize Met231 and Met250. The disappearance or the change of intensity of some peaks tells us that we are in a slow-exchange situation.

Chemical shift differences of Met 231 resonances between the free vs Compound (**50**) bound form provide an estimated upper limit for the off rate for the complex of $k_{\mathrm{off}} < 519\ \mathrm{s}^{-1}$, that assuming a diffusion limited on the rate of $10^9\ \mathrm{M}^{-1}\ \mathrm{s}^{-1}$, would correspond to a low micromolar dissociation constant K_{d}.

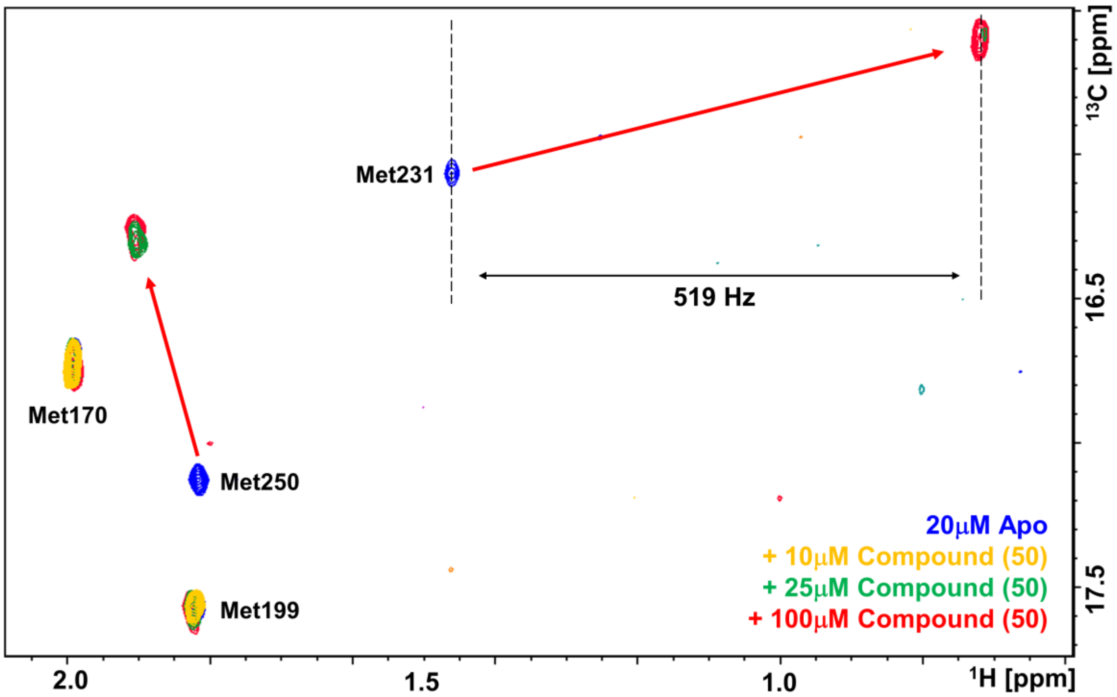

Figure S10. HPLC trace Compound (**21**) (purity > 98%).

Additional Info : Peak(s) manually integrated

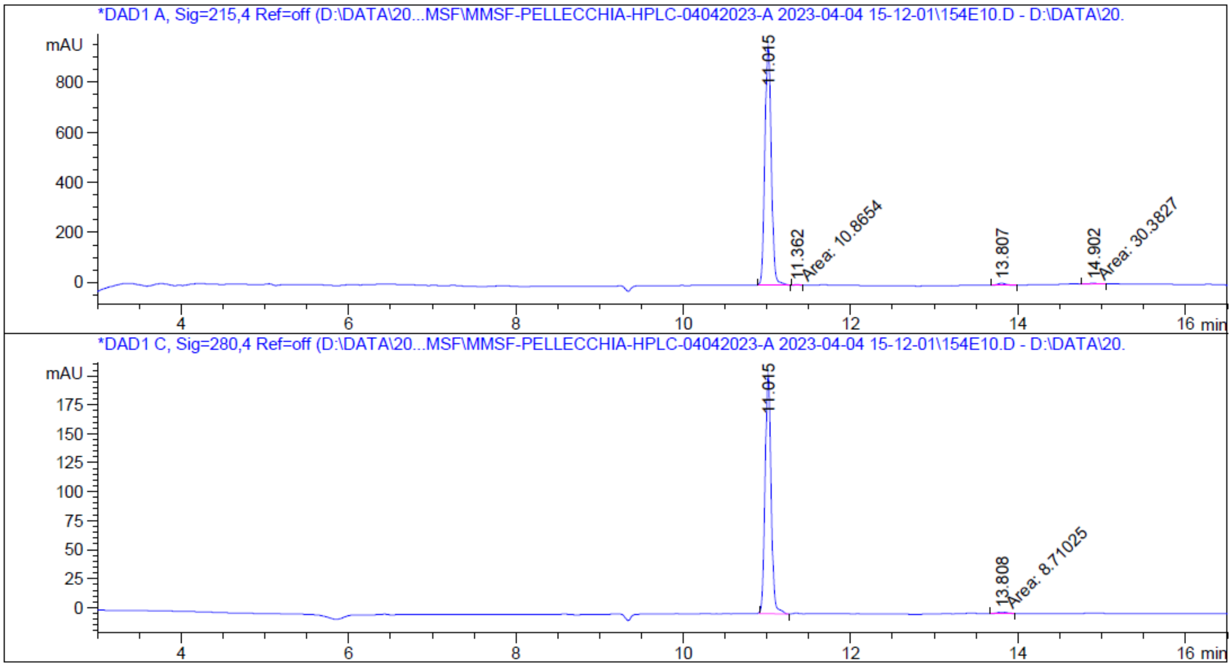

Area Percent Report

Sorted By : Signal
Multiplier: : 1.0000
Dilution: : 1.0000
Use Multiplier & Dilution Factor with ISTDs

Signal 1: DAD1 A, Sig=215,4 Ref=off
Signal has been modified after loading from rawdata file!

Peak #	RetTime [min]	Type	Width [min]	Area [mAU*s]	Height [mAU]	Area %
1	11.015	BV	0.0843	5096.64990	955.03149	98.2663
2	11.362	MM	0.0778	10.86542	2.32901	0.2095

Peak #	RetTime [min]	Type	Width [min]	Area [mAU*s]	Height [mAU]	Area %
3	13.807	BB	0.0960	48.67250	7.88320	0.9384
4	14.902	MM	0.1670	30.38273	2.23821	0.5858

Figure S11. HPLC trace for Compound (**50**) (peak 1) (purity > 99 %).

Additional Info : Peak(s) manually integrated

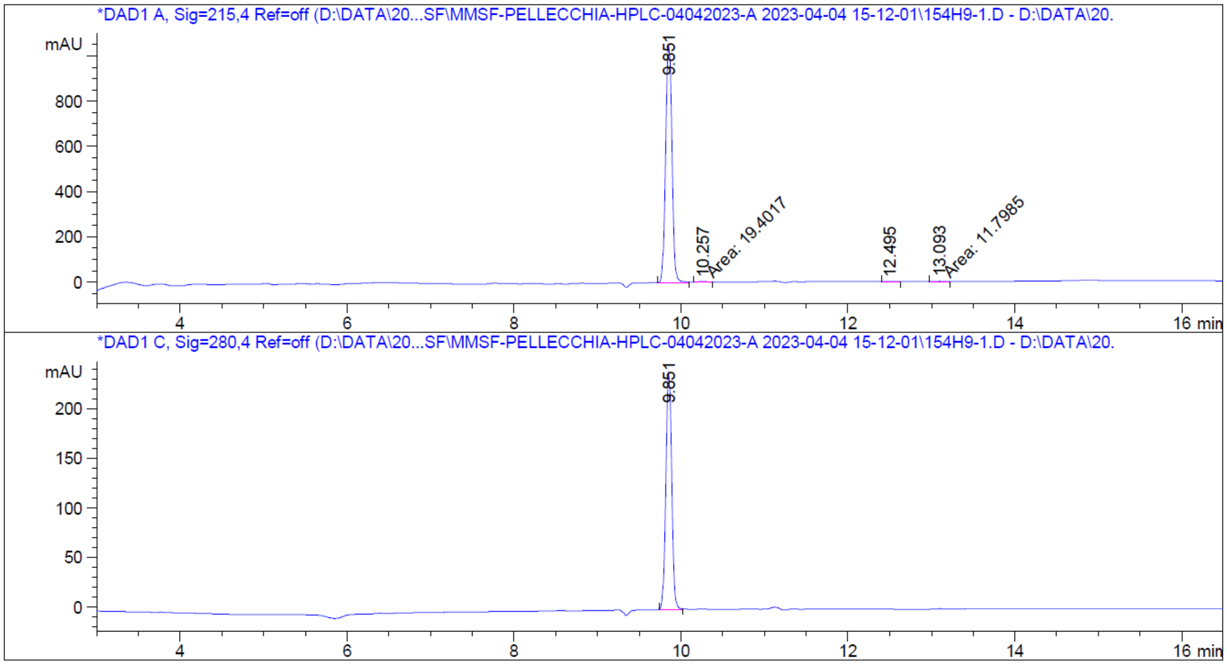

===
Area Percent Report
===

Sorted By : Signal
Multiplier: : 1.0000
Dilution: : 1.0000
Use Multiplier & Dilution Factor with ISTDs

Signal 1: DAD1 A, Sig=215,4 Ref=off
Signal has been modified after loading from rawdata file!

Peak #	RetTime [min]	Type	Width [min]	Area [mAU*s]	Height [mAU]	Area %
1	9.851	BB	0.0839	5557.36230	1047.73254	99.2718
2	10.257	MM	0.1136	19.40169	2.84659	0.3466

Instrument 1 4/4/2023 5:45:10 PM Page 1 of 2

Data File D:\DATA\2023\MMSF\MMSF-PELLECCHIA-HPLC-04042023-A 2023-04-04 15-12-01\154H9-1.D
Sample Name: 154H9-1

Peak #	RetTime [min]	Type	Width [min]	Area [mAU*s]	Height [mAU]	Area %
3	12.495	BB	0.0822	9.56795	1.79358	0.1709
4	13.093	MM	0.1269	11.79854	1.55009	0.2108

Figure S12. HPLC trace for Compound (**51**) (peak 2) (purity ~ 95 %).

Additional Info : Peak(s) manually integrated

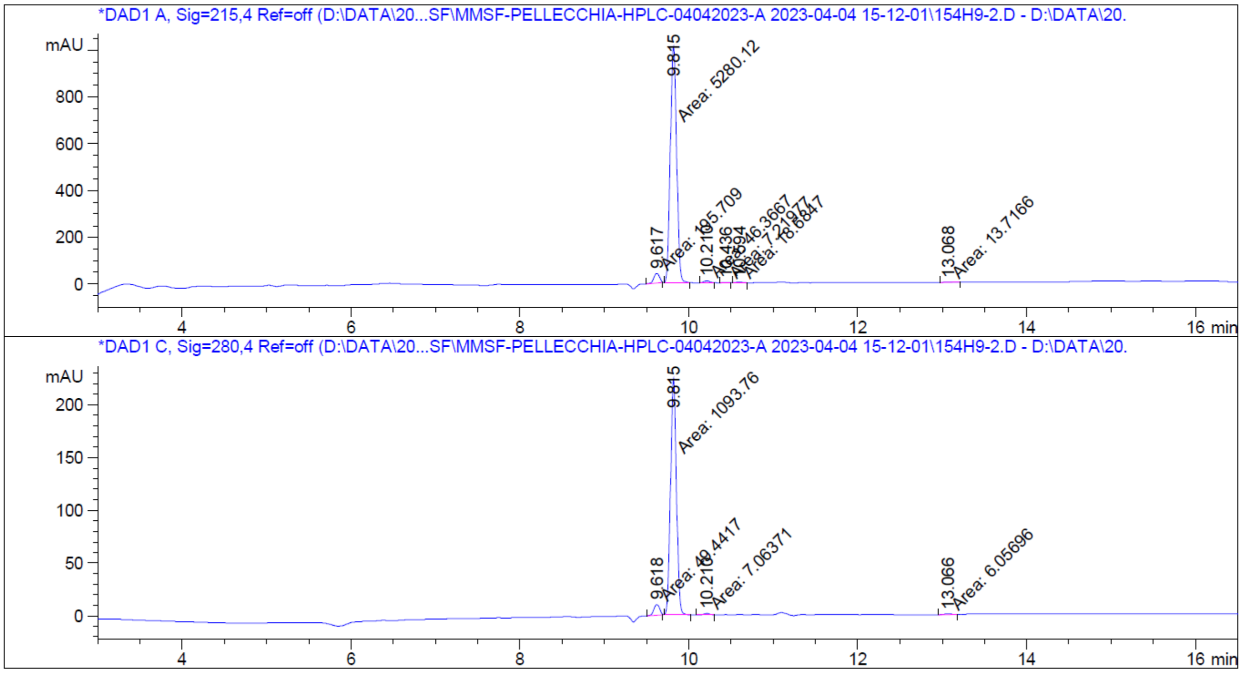


```
==================================================================
                        Area Percent Report
==================================================================

Sorted By             :      Signal
Multiplier:                  :       1.0000
Dilution:                    :       1.0000
Use Multiplier & Dilution Factor with ISTDs


Signal 1: DAD1 A, Sig=215,4 Ref=off
 Signal has been modified after loading from rawdata file!
```

Peak #	RetTime [min]	Type	Width [min]	Area [mAU*s]	Height [mAU]	Area %
1	9.617	MM	0.0795	195.70894	41.02414	3.5188
2	9.815	MM	0.0867	5280.12402	1014.51263	94.9352

Data File D:\DATA\2023\MMSF\MMSF-PELLECCHIA-HPLC-04042023-A 2023-04-04 15-12-01\154H9-2.D
Sample Name: 154H9-2

Peak #	RetTime [min]	Type	Width [min]	Area [mAU*s]	Height [mAU]	Area %
3	10.210	MM	0.0862	46.36669	8.96749	0.8337
4	10.436	MM	0.0778	7.21977	1.54661	0.1298
5	10.594	MM	0.0829	18.68472	3.75796	0.3359
6	13.068	MM	0.1066	13.71661	2.14457	0.2466
